# Measurement of the photon identification efficiencies with the ATLAS detector using LHC Run-1 data

**DOI:** 10.1140/epjc/s10052-016-4507-9

**Published:** 2016-12-03

**Authors:** M. Aaboud, G. Aad, B. Abbott, J. Abdallah, O. Abdinov, B. Abeloos, R. Aben, O. S. AbouZeid, N. L. Abraham, H. Abramowicz, H. Abreu, R. Abreu, Y. Abulaiti, B. S. Acharya, L. Adamczyk, D. L. Adams, J. Adelman, S. Adomeit, T. Adye, A. A. Affolder, T. Agatonovic-Jovin, J. Agricola, J. A. Aguilar-Saavedra, S. P. Ahlen, F. Ahmadov, G. Aielli, H. Akerstedt, T. P. A. Åkesson, A. V. Akimov, G. L. Alberghi, J. Albert, S. Albrand, M. J. Alconada Verzini, M. Aleksa, I. N. Aleksandrov, C. Alexa, G. Alexander, T. Alexopoulos, M. Alhroob, M. Aliev, G. Alimonti, J. Alison, S. P. Alkire, B. M. M. Allbrooke, B. W. Allen, P. P. Allport, A. Aloisio, A. Alonso, F. Alonso, C. Alpigiani, M. Alstaty, B. Alvarez Gonzalez, D. Álvarez Piqueras, M. G. Alviggi, B. T. Amadio, K. Amako, Y. Amaral Coutinho, C. Amelung, D. Amidei, S. P. Amor Dos Santos, A. Amorim, S. Amoroso, G. Amundsen, C. Anastopoulos, L. S. Ancu, N. Andari, T. Andeen, C. F. Anders, G. Anders, J. K. Anders, K. J. Anderson, A. Andreazza, V. Andrei, S. Angelidakis, I. Angelozzi, P. Anger, A. Angerami, F. Anghinolfi, A. V. Anisenkov, N. Anjos, A. Annovi, M. Antonelli, A. Antonov, F. Anulli, M. Aoki, L. Aperio Bella, G. Arabidze, Y. Arai, J. P. Araque, A. T. H. Arce, F. A. Arduh, J.-F. Arguin, S. Argyropoulos, M. Arik, A. J. Armbruster, L. J. Armitage, O. Arnaez, H. Arnold, M. Arratia, O. Arslan, A. Artamonov, G. Artoni, S. Artz, S. Asai, N. Asbah, A. Ashkenazi, B. Åsman, L. Asquith, K. Assamagan, R. Astalos, M. Atkinson, N. B. Atlay, K. Augsten, G. Avolio, B. Axen, M. K. Ayoub, G. Azuelos, M. A. Baak, A. E. Baas, M. J. Baca, H. Bachacou, K. Bachas, M. Backes, M. Backhaus, P. Bagiacchi, P. Bagnaia, Y. Bai, J. T. Baines, O. K. Baker, E. M. Baldin, P. Balek, T. Balestri, F. Balli, W. K. Balunas, E. Banas, Sw. Banerjee, A. A. E. Bannoura, L. Barak, E. L. Barberio, D. Barberis, M. Barbero, T. Barillari, T. Barklow, N. Barlow, S. L. Barnes, B. M. Barnett, R. M. Barnett, Z. Barnovska, A. Baroncelli, G. Barone, A. J. Barr, L. Barranco Navarro, F. Barreiro, J. Barreiro Guimarães da Costa, R. Bartoldus, A. E. Barton, P. Bartos, A. Basalaev, A. Bassalat, R. L. Bates, S. J. Batista, J. R. Batley, M. Battaglia, M. Bauce, F. Bauer, H. S. Bawa, J. B. Beacham, M. D. Beattie, T. Beau, P. H. Beauchemin, P. Bechtle, H. P. Beck, K. Becker, M. Becker, M. Beckingham, C. Becot, A. J. Beddall, A. Beddall, V. A. Bednyakov, M. Bedognetti, C. P. Bee, L. J. Beemster, T. A. Beermann, M. Begel, J. K. Behr, C. Belanger-Champagne, A. S. Bell, G. Bella, L. Bellagamba, A. Bellerive, M. Bellomo, K. Belotskiy, O. Beltramello, N. L. Belyaev, O. Benary, D. Benchekroun, M. Bender, K. Bendtz, N. Benekos, Y. Benhammou, E. Benhar Noccioli, J. Benitez, D. P. Benjamin, J. R. Bensinger, S. Bentvelsen, L. Beresford, M. Beretta, D. Berge, E. Bergeaas Kuutmann, N. Berger, J. Beringer, S. Berlendis, N. R. Bernard, C. Bernius, F. U. Bernlochner, T. Berry, P. Berta, C. Bertella, G. Bertoli, F. Bertolucci, I. A. Bertram, C. Bertsche, D. Bertsche, G. J. Besjes, O. Bessidskaia Bylund, M. Bessner, N. Besson, C. Betancourt, S. Bethke, A. J. Bevan, W. Bhimji, R. M. Bianchi, L. Bianchini, M. Bianco, O. Biebel, D. Biedermann, R. Bielski, N. V. Biesuz, M. Biglietti, J. Bilbao De Mendizabal, H. Bilokon, M. Bindi, S. Binet, A. Bingul, C. Bini, S. Biondi, D. M. Bjergaard, C. W. Black, J. E. Black, K. M. Black, D. Blackburn, R. E. Blair, J.-B. Blanchard, J. E. Blanco, T. Blazek, I. Bloch, C. Blocker, W. Blum, U. Blumenschein, S. Blunier, G. J. Bobbink, V. S. Bobrovnikov, S. S. Bocchetta, A. Bocci, C. Bock, M. Boehler, D. Boerner, J. A. Bogaerts, D. Bogavac, A. G. Bogdanchikov, C. Bohm, V. Boisvert, P. Bokan, T. Bold, A. S. Boldyrev, M. Bomben, M. Bona, M. Boonekamp, A. Borisov, G. Borissov, J. Bortfeldt, D. Bortoletto, V. Bortolotto, K. Bos, D. Boscherini, M. Bosman, J. D. Bossio Sola, J. Boudreau, J. Bouffard, E. V. Bouhova-Thacker, D. Boumediene, C. Bourdarios, S. K. Boutle, A. Boveia, J. Boyd, I. R. Boyko, J. Bracinik, A. Brandt, G. Brandt, O. Brandt, U. Bratzler, B. Brau, J. E. Brau, H. M. Braun, W. D. Breaden Madden, K. Brendlinger, A. J. Brennan, L. Brenner, R. Brenner, S. Bressler, T. M. Bristow, D. Britton, D. Britzger, F. M. Brochu, I. Brock, R. Brock, G. Brooijmans, T. Brooks, W. K. Brooks, J. Brosamer, E. Brost, J. H Broughton, P. A. Bruckman de Renstrom, D. Bruncko, R. Bruneliere, A. Bruni, G. Bruni, L. S. Bruni, B. H. Brunt, M. Bruschi, N. Bruscino, P. Bryant, L. Bryngemark, T. Buanes, Q. Buat, P. Buchholz, A. G. Buckley, I. A. Budagov, F. Buehrer, M. K. Bugge, O. Bulekov, D. Bullock, H. Burckhart, S. Burdin, C. D. Burgard, B. Burghgrave, K. Burka, S. Burke, I. Burmeister, E. Busato, D. Büscher, V. Büscher, P. Bussey, J. M. Butler, C. M. Buttar, J. M. Butterworth, P. Butti, W. Buttinger, A. Buzatu, A. R. Buzykaev, S. Cabrera Urbán, D. Caforio, V. M. Cairo, O. Cakir, N. Calace, P. Calafiura, A. Calandri, G. Calderini, P. Calfayan, L. P. Caloba, D. Calvet, S. Calvet, T. P. Calvet, R. Camacho Toro, S. Camarda, P. Camarri, D. Cameron, R. Caminal Armadans, C. Camincher, S. Campana, M. Campanelli, A. Camplani, A. Campoverde, V. Canale, A. Canepa, M. Cano Bret, J. Cantero, R. Cantrill, T. Cao, M. D. M. Capeans Garrido, I. Caprini, M. Caprini, M. Capua, R. Caputo, R. M. Carbone, R. Cardarelli, F. Cardillo, I. Carli, T. Carli, G. Carlino, L. Carminati, S. Caron, E. Carquin, G. D. Carrillo-Montoya, J. R. Carter, J. Carvalho, D. Casadei, M. P. Casado, M. Casolino, D. W. Casper, E. Castaneda-Miranda, R. Castelijn, A. Castelli, V. Castillo Gimenez, N. F. Castro, A. Catinaccio, J. R. Catmore, A. Cattai, J. Caudron, V. Cavaliere, E. Cavallaro, D. Cavalli, M. Cavalli-Sforza, V. Cavasinni, F. Ceradini, L. Cerda Alberich, B. C. Cerio, A. S. Cerqueira, A. Cerri, L. Cerrito, F. Cerutti, M. Cerv, A. Cervelli, S. A. Cetin, A. Chafaq, D. Chakraborty, S. K. Chan, Y. L. Chan, P. Chang, J. D. Chapman, D. G. Charlton, A. Chatterjee, C. C. Chau, C. A. Chavez Barajas, S. Che, S. Cheatham, A. Chegwidden, S. Chekanov, S. V. Chekulaev, G. A. Chelkov, M. A. Chelstowska, C. Chen, H. Chen, K. Chen, S. Chen, S. Chen, X. Chen, Y. Chen, H. C. Cheng, H. J. Cheng, Y. Cheng, A. Cheplakov, E. Cheremushkina, R. Cherkaoui El Moursli, V. Chernyatin, E. Cheu, L. Chevalier, V. Chiarella, G. Chiarelli, G. Chiodini, A. S. Chisholm, A. Chitan, M. V. Chizhov, K. Choi, A. R. Chomont, S. Chouridou, B. K. B. Chow, V. Christodoulou, D. Chromek-Burckhart, J. Chudoba, A. J. Chuinard, J. J. Chwastowski, L. Chytka, G. Ciapetti, A. K. Ciftci, D. Cinca, V. Cindro, I. A. Cioara, A. Ciocio, F. Cirotto, Z. H. Citron, M. Citterio, M. Ciubancan, A. Clark, B. L. Clark, M. R. Clark, P. J. Clark, R. N. Clarke, C. Clement, Y. Coadou, M. Cobal, A. Coccaro, J. Cochran, L. Coffey, L. Colasurdo, B. Cole, A. P. Colijn, J. Collot, T. Colombo, G. Compostella, P. Conde Muiño, E. Coniavitis, S. H. Connell, I. A. Connelly, V. Consorti, S. Constantinescu, G. Conti, F. Conventi, M. Cooke, B. D. Cooper, A. M. Cooper-Sarkar, K. J. R. Cormier, T. Cornelissen, M. Corradi, F. Corriveau, A. Corso-Radu, A. Cortes-Gonzalez, G. Cortiana, G. Costa, M. J. Costa, D. Costanzo, G. Cottin, G. Cowan, B. E. Cox, K. Cranmer, S. J. Crawley, G. Cree, S. Crépé-Renaudin, F. Crescioli, W. A. Cribbs, M. Crispin Ortuzar, M. Cristinziani, V. Croft, G. Crosetti, T. Cuhadar Donszelmann, J. Cummings, M. Curatolo, J. Cúth, C. Cuthbert, H. Czirr, P. Czodrowski, G. D’amen, S. D’Auria, M. D’Onofrio, M. J. Da Cunha Sargedas De Sousa, C. Da Via, W. Dabrowski, T. Dado, T. Dai, O. Dale, F. Dallaire, C. Dallapiccola, M. Dam, J. R. Dandoy, N. P. Dang, A. C. Daniells, N. S. Dann, M. Danninger, M. Dano Hoffmann, V. Dao, G. Darbo, S. Darmora, J. Dassoulas, A. Dattagupta, W. Davey, C. David, T. Davidek, M. Davies, P. Davison, E. Dawe, I. Dawson, R. K. Daya-Ishmukhametova, K. De, R. de Asmundis, A. De Benedetti, S. De Castro, S. De Cecco, N. De Groot, P. de Jong, H. De la Torre, F. De Lorenzi, A. De Maria, D. De Pedis, A. De Salvo, U. De Sanctis, A. De Santo, J. B. De Vivie De Regie, W. J. Dearnaley, R. Debbe, C. Debenedetti, D. V. Dedovich, N. Dehghanian, I. Deigaard, M. Del Gaudio, J. Del Peso, T. Del Prete, D. Delgove, F. Deliot, C. M. Delitzsch, M. Deliyergiyev, A. Dell’Acqua, L. Dell’Asta, M. Dell’Orso, M. Della Pietra, D. della Volpe, M. Delmastro, P. A. Delsart, C. Deluca, D. A. DeMarco, S. Demers, M. Demichev, A. Demilly, S. P. Denisov, D. Denysiuk, D. Derendarz, J. E. Derkaoui, F. Derue, P. Dervan, K. Desch, C. Deterre, K. Dette, P. O. Deviveiros, A. Dewhurst, S. Dhaliwal, A. Di Ciaccio, L. Di Ciaccio, W. K. Di Clemente, C. Di Donato, A. Di Girolamo, B. Di Girolamo, B. Di Micco, R. Di Nardo, A. Di Simone, R. Di Sipio, D. Di Valentino, C. Diaconu, M. Diamond, F. A. Dias, M. A. Diaz, E. B. Diehl, J. Dietrich, S. Diglio, A. Dimitrievska, J. Dingfelder, P. Dita, S. Dita, F. Dittus, F. Djama, T. Djobava, J. I. Djuvsland, M. A. B. do Vale, D. Dobos, M. Dobre, C. Doglioni, T. Dohmae, J. Dolejsi, Z. Dolezal, B. A. Dolgoshein, M. Donadelli, S. Donati, P. Dondero, J. Donini, J. Dopke, A. Doria, M. T. Dova, A. T. Doyle, E. Drechsler, M. Dris, Y. Du, J. Duarte-Campderros, E. Duchovni, G. Duckeck, O. A. Ducu, D. Duda, A. Dudarev, E. M. Duffield, L. Duflot, L. Duguid, M. Dührssen, M. Dumancic, M. Dunford, H. Duran Yildiz, M. Düren, A. Durglishvili, D. Duschinger, B. Dutta, M. Dyndal, C. Eckardt, K. M. Ecker, R. C. Edgar, N. C. Edwards, T. Eifert, G. Eigen, K. Einsweiler, T. Ekelof, M. El Kacimi, V. Ellajosyula, M. Ellert, S. Elles, F. Ellinghaus, A. A. Elliot, N. Ellis, J. Elmsheuser, M. Elsing, D. Emeliyanov, Y. Enari, O. C. Endner, M. Endo, J. S. Ennis, J. Erdmann, A. Ereditato, G. Ernis, J. Ernst, M. Ernst, S. Errede, E. Ertel, M. Escalier, H. Esch, C. Escobar, B. Esposito, A. I. Etienvre, E. Etzion, H. Evans, A. Ezhilov, F. Fabbri, L. Fabbri, G. Facini, R. M. Fakhrutdinov, S. Falciano, R. J. Falla, J. Faltova, Y. Fang, M. Fanti, A. Farbin, A. Farilla, C. Farina, T. Farooque, S. Farrell, S. M. Farrington, P. Farthouat, F. Fassi, P. Fassnacht, D. Fassouliotis, M. Faucci Giannelli, A. Favareto, W. J. Fawcett, L. Fayard, O. L. Fedin, W. Fedorko, S. Feigl, L. Feligioni, C. Feng, E. J. Feng, H. Feng, A. B. Fenyuk, L. Feremenga, P. Fernandez Martinez, S. Fernandez Perez, J. Ferrando, A. Ferrari, P. Ferrari, R. Ferrari, D. E. Ferreira de Lima, A. Ferrer, D. Ferrere, C. Ferretti, A. Ferretto Parodi, F. Fiedler, A. Filipčič, M. Filipuzzi, F. Filthaut, M. Fincke-Keeler, K. D. Finelli, M. C. N. Fiolhais, L. Fiorini, A. Firan, A. Fischer, C. Fischer, J. Fischer, W. C. Fisher, N. Flaschel, I. Fleck, P. Fleischmann, G. T. Fletcher, R. R. M. Fletcher, T. Flick, A. Floderus, L. R. Flores Castillo, M. J. Flowerdew, G. T. Forcolin, A. Formica, A. Forti, A. G. Foster, D. Fournier, H. Fox, S. Fracchia, P. Francavilla, M. Franchini, D. Francis, L. Franconi, M. Franklin, M. Frate, M. Fraternali, D. Freeborn, S. M. Fressard-Batraneanu, F. Friedrich, D. Froidevaux, J. A. Frost, C. Fukunaga, E. Fullana Torregrosa, T. Fusayasu, J. Fuster, C. Gabaldon, O. Gabizon, A. Gabrielli, A. Gabrielli, G. P. Gach, S. Gadatsch, S. Gadomski, G. Gagliardi, L. G. Gagnon, P. Gagnon, C. Galea, B. Galhardo, E. J. Gallas, B. J. Gallop, P. Gallus, G. Galster, K. K. Gan, J. Gao, Y. Gao, Y. S. Gao, F. M. Garay Walls, C. García, J. E. García Navarro, M. Garcia-Sciveres, R. W. Gardner, N. Garelli, V. Garonne, A. Gascon Bravo, C. Gatti, A. Gaudiello, G. Gaudio, B. Gaur, L. Gauthier, I. L. Gavrilenko, C. Gay, G. Gaycken, E. N. Gazis, Z. Gecse, C. N. P. Gee, Ch. Geich-Gimbel, M. Geisen, M. P. Geisler, C. Gemme, M. H. Genest, C. Geng, S. Gentile, S. George, D. Gerbaudo, A. Gershon, S. Ghasemi, H. Ghazlane, M. Ghneimat, B. Giacobbe, S. Giagu, P. Giannetti, B. Gibbard, S. M. Gibson, M. Gignac, M. Gilchriese, T. P. S. Gillam, D. Gillberg, G. Gilles, D. M. Gingrich, N. Giokaris, M. P. Giordani, F. M. Giorgi, F. M. Giorgi, P. F. Giraud, P. Giromini, D. Giugni, F. Giuli, C. Giuliani, M. Giulini, B. K. Gjelsten, S. Gkaitatzis, I. Gkialas, E. L. Gkougkousis, L. K. Gladilin, C. Glasman, J. Glatzer, P. C. F. Glaysher, A. Glazov, M. Goblirsch-Kolb, J. Godlewski, S. Goldfarb, T. Golling, D. Golubkov, A. Gomes, R. Gonçalo, J. Goncalves Pinto Firmino Da Costa, G. Gonella, L. Gonella, A. Gongadze, S. González de la Hoz, G. Gonzalez Parra, S. Gonzalez-Sevilla, L. Goossens, P. A. Gorbounov, H. A. Gordon, I. Gorelov, B. Gorini, E. Gorini, A. Gorišek, E. Gornicki, A. T. Goshaw, C. Gössling, M. I. Gostkin, C. R. Goudet, D. Goujdami, A. G. Goussiou, N. Govender, E. Gozani, L. Graber, I. Grabowska-Bold, P. O. J. Gradin, P. Grafström, J. Gramling, E. Gramstad, S. Grancagnolo, V. Gratchev, P. M. Gravila, H. M. Gray, E. Graziani, Z. D. Greenwood, C. Grefe, K. Gregersen, I. M. Gregor, P. Grenier, K. Grevtsov, J. Griffiths, A. A. Grillo, K. Grimm, S. Grinstein, Ph. Gris, J.-F. Grivaz, S. Groh, J. P. Grohs, E. Gross, J. Grosse-Knetter, G. C. Grossi, Z. J. Grout, L. Guan, W. Guan, J. Guenther, F. Guescini, D. Guest, O. Gueta, E. Guido, T. Guillemin, S. Guindon, U. Gul, C. Gumpert, J. Guo, Y. Guo, S. Gupta, G. Gustavino, P. Gutierrez, N. G. Gutierrez Ortiz, C. Gutschow, C. Guyot, C. Gwenlan, C. B. Gwilliam, A. Haas, C. Haber, H. K. Hadavand, N. Haddad, A. Hadef, P. Haefner, S. Hageböck, Z. Hajduk, H. Hakobyan, M. Haleem, J. Haley, G. Halladjian, G. D. Hallewell, K. Hamacher, P. Hamal, K. Hamano, A. Hamilton, G. N. Hamity, P. G. Hamnett, L. Han, K. Hanagaki, K. Hanawa, M. Hance, B. Haney, P. Hanke, R. Hanna, J. B. Hansen, J. D. Hansen, M. C. Hansen, P. H. Hansen, K. Hara, A. S. Hard, T. Harenberg, F. Hariri, S. Harkusha, R. D. Harrington, P. F. Harrison, F. Hartjes, N. M. Hartmann, M. Hasegawa, Y. Hasegawa, A. Hasib, S. Hassani, S. Haug, R. Hauser, L. Hauswald, M. Havranek, C. M. Hawkes, R. J. Hawkings, D. Hayden, C. P. Hays, J. M. Hays, H. S. Hayward, S. J. Haywood, S. J. Head, T. Heck, V. Hedberg, L. Heelan, S. Heim, T. Heim, B. Heinemann, J. J. Heinrich, L. Heinrich, C. Heinz, J. Hejbal, L. Helary, S. Hellman, C. Helsens, J. Henderson, R. C. W. Henderson, Y. Heng, S. Henkelmann, A. M. Henriques Correia, S. Henrot-Versille, G. H. Herbert, Y. Hernández Jiménez, G. Herten, R. Hertenberger, L. Hervas, G. G. Hesketh, N. P. Hessey, J. W. Hetherly, R. Hickling, E. Higón-Rodriguez, E. Hill, J. C. Hill, K. H. Hiller, S. J. Hillier, I. Hinchliffe, E. Hines, R. R. Hinman, M. Hirose, D. Hirschbuehl, J. Hobbs, N. Hod, M. C. Hodgkinson, P. Hodgson, A. Hoecker, M. R. Hoeferkamp, F. Hoenig, D. Hohn, T. R. Holmes, M. Homann, T. M. Hong, B. H. Hooberman, W. H. Hopkins, Y. Horii, A. J. Horton, J.-Y. Hostachy, S. Hou, A. Hoummada, J. Howarth, M. Hrabovsky, I. Hristova, J. Hrivnac, T. Hryn’ova, A. Hrynevich, C. Hsu, P. J. Hsu, S.-C. Hsu, D. Hu, Q. Hu, Y. Huang, Z. Hubacek, F. Hubaut, F. Huegging, T. B. Huffman, E. W. Hughes, G. Hughes, M. Huhtinen, T. A. Hülsing, P. Huo, N. Huseynov, J. Huston, J. Huth, G. Iacobucci, G. Iakovidis, I. Ibragimov, L. Iconomidou-Fayard, E. Ideal, Z. Idrissi, P. Iengo, O. Igonkina, T. Iizawa, Y. Ikegami, M. Ikeno, Y. Ilchenko, D. Iliadis, N. Ilic, T. Ince, G. Introzzi, P. Ioannou, M. Iodice, K. Iordanidou, V. Ippolito, M. Ishino, M. Ishitsuka, R. Ishmukhametov, C. Issever, S. Istin, F. Ito, J. M. Iturbe Ponce, R. Iuppa, W. Iwanski, H. Iwasaki, J. M. Izen, V. Izzo, S. Jabbar, B. Jackson, M. Jackson, P. Jackson, V. Jain, K. B. Jakobi, K. Jakobs, S. Jakobsen, T. Jakoubek, D. O. Jamin, D. K. Jana, E. Jansen, R. Jansky, J. Janssen, M. Janus, G. Jarlskog, N. Javadov, T. Javůrek, F. Jeanneau, L. Jeanty, J. Jejelava, G.-Y. Jeng, D. Jennens, P. Jenni, J. Jentzsch, C. Jeske, S. Jézéquel, H. Ji, J. Jia, H. Jiang, Y. Jiang, S. Jiggins, M. Jimenez Belenguer, J. Jimenez Pena, S. Jin, A. Jinaru, O. Jinnouchi, P. Johansson, K. A. Johns, W. J. Johnson, K. Jon-And, G. Jones, R. W. L. Jones, S. Jones, T. J. Jones, J. Jongmanns, P. M. Jorge, J. Jovicevic, X. Ju, A. Juste Rozas, M. K. Köhler, A. Kaczmarska, M. Kado, H. Kagan, M. Kagan, S. J. Kahn, E. Kajomovitz, C. W. Kalderon, A. Kaluza, S. Kama, A. Kamenshchikov, N. Kanaya, S. Kaneti, L. Kanjir, V. A. Kantserov, J. Kanzaki, B. Kaplan, L. S. Kaplan, A. Kapliy, D. Kar, K. Karakostas, A. Karamaoun, N. Karastathis, M. J. Kareem, E. Karentzos, M. Karnevskiy, S. N. Karpov, Z. M. Karpova, K. Karthik, V. Kartvelishvili, A. N. Karyukhin, K. Kasahara, L. Kashif, R. D. Kass, A. Kastanas, Y. Kataoka, C. Kato, A. Katre, J. Katzy, K. Kawagoe, T. Kawamoto, G. Kawamura, S. Kazama, V. F. Kazanin, R. Keeler, R. Kehoe, J. S. Keller, J. J. Kempster, K. Kawade, H. Keoshkerian, O. Kepka, B. P. Kerševan, S. Kersten, R. A. Keyes, F. Khalil-zada, A. Khanov, A. G. Kharlamov, T. J. Khoo, V. Khovanskiy, E. Khramov, J. Khubua, S. Kido, H. Y. Kim, S. H. Kim, Y. K. Kim, N. Kimura, O. M. Kind, B. T. King, M. King, S. B. King, J. Kirk, A. E. Kiryunin, T. Kishimoto, D. Kisielewska, F. Kiss, K. Kiuchi, O. Kivernyk, E. Kladiva, M. H. Klein, M. Klein, U. Klein, K. Kleinknecht, P. Klimek, A. Klimentov, R. Klingenberg, J. A. Klinger, T. Klioutchnikova, E.-E. Kluge, P. Kluit, S. Kluth, J. Knapik, E. Kneringer, E. B. F. G. Knoops, A. Knue, A. Kobayashi, D. Kobayashi, T. Kobayashi, M. Kobel, M. Kocian, P. Kodys, T. Koffas, E. Koffeman, T. Koi, H. Kolanoski, M. Kolb, I. Koletsou, A. A. Komar, Y. Komori, T. Kondo, N. Kondrashova, K. Köneke, A. C. König, T. Kono, R. Konoplich, N. Konstantinidis, R. Kopeliansky, S. Koperny, L. Köpke, A. K. Kopp, K. Korcyl, K. Kordas, A. Korn, A. A. Korol, I. Korolkov, E. V. Korolkova, O. Kortner, S. Kortner, T. Kosek, V. V. Kostyukhin, A. Kotwal, A. Kourkoumeli-Charalampidi, C. Kourkoumelis, V. Kouskoura, A. B. Kowalewska, R. Kowalewski, T. Z. Kowalski, C. Kozakai, W. Kozanecki, A. S. Kozhin, V. A. Kramarenko, G. Kramberger, D. Krasnopevtsev, M. W. Krasny, A. Krasznahorkay, J. K. Kraus, A. Kravchenko, M. Kretz, J. Kretzschmar, K. Kreutzfeldt, P. Krieger, K. Krizka, K. Kroeninger, H. Kroha, J. Kroll, J. Kroseberg, J. Krstic, U. Kruchonak, H. Krüger, N. Krumnack, A. Kruse, M. C. Kruse, M. Kruskal, T. Kubota, H. Kucuk, S. Kuday, J. T. Kuechler, S. Kuehn, A. Kugel, F. Kuger, A. Kuhl, T. Kuhl, V. Kukhtin, R. Kukla, Y. Kulchitsky, S. Kuleshov, M. Kuna, T. Kunigo, A. Kupco, H. Kurashige, Y. A. Kurochkin, V. Kus, E. S. Kuwertz, M. Kuze, J. Kvita, T. Kwan, D. Kyriazopoulos, A. La Rosa, J. L. La Rosa Navarro, L. La Rotonda, C. Lacasta, F. Lacava, J. Lacey, H. Lacker, D. Lacour, V. R. Lacuesta, E. Ladygin, R. Lafaye, B. Laforge, T. Lagouri, S. Lai, S. Lammers, W. Lampl, E. Lançon, U. Landgraf, M. P. J. Landon, V. S. Lang, J. C. Lange, A. J. Lankford, F. Lanni, K. Lantzsch, A. Lanza, S. Laplace, C. Lapoire, J. F. Laporte, T. Lari, F. Lasagni Manghi, M. Lassnig, P. Laurelli, W. Lavrijsen, A. T. Law, P. Laycock, T. Lazovich, M. Lazzaroni, B. Le, O. Le Dortz, E. Le Guirriec, E. P. Le Quilleuc, M. LeBlanc, T. LeCompte, F. Ledroit-Guillon, C. A. Lee, S. C. Lee, L. Lee, G. Lefebvre, M. Lefebvre, F. Legger, C. Leggett, A. Lehan, G. Lehmann Miotto, X. Lei, W. A. Leight, A. Leisos, A. G. Leister, M. A. L. Leite, R. Leitner, D. Lellouch, B. Lemmer, K. J. C. Leney, T. Lenz, B. Lenzi, R. Leone, S. Leone, C. Leonidopoulos, S. Leontsinis, G. Lerner, C. Leroy, A. A. J. Lesage, C. G. Lester, M. Levchenko, J. Levêque, D. Levin, L. J. Levinson, M. Levy, D. Lewis, A. M. Leyko, M. Leyton, B. Li, H. Li, H. L. Li, L. Li, L. Li, Q. Li, S. Li, X. Li, Y. Li, Z. Liang, B. Liberti, A. Liblong, P. Lichard, K. Lie, J. Liebal, W. Liebig, A. Limosani, S. C. Lin, T. H. Lin, B. E. Lindquist, A. E. Lionti, E. Lipeles, A. Lipniacka, M. Lisovyi, T. M. Liss, A. Lister, A. M. Litke, B. Liu, D. Liu, H. Liu, H. Liu, J. Liu, J. B. Liu, K. Liu, L. Liu, M. Liu, M. Liu, Y. L. Liu, Y. Liu, M. Livan, A. Lleres, J. Llorente Merino, S. L. Lloyd, F. Lo Sterzo, E. Lobodzinska, P. Loch, W. S. Lockman, F. K. Loebinger, A. E. Loevschall-Jensen, K. M. Loew, A. Loginov, T. Lohse, K. Lohwasser, M. Lokajicek, B. A. Long, J. D. Long, R. E. Long, L. Longo, K. A. Looper, L. Lopes, D. Lopez Mateos, B. Lopez Paredes, I. Lopez Paz, A. Lopez Solis, J. Lorenz, N. Lorenzo Martinez, M. Losada, P. J. Lösel, X. Lou, A. Lounis, J. Love, P. A. Love, H. Lu, N. Lu, H. J. Lubatti, C. Luci, A. Lucotte, C. Luedtke, F. Luehring, W. Lukas, L. Luminari, O. Lundberg, B. Lund-Jensen, P. M. Luzi, D. Lynn, R. Lysak, E. Lytken, V. Lyubushkin, H. Ma, L. L. Ma, Y. Ma, G. Maccarrone, A. Macchiolo, C. M. Macdonald, B. Maček, J. Machado Miguens, D. Madaffari, R. Madar, H. J. Maddocks, W. F. Mader, A. Madsen, J. Maeda, S. Maeland, T. Maeno, A. Maevskiy, E. Magradze, J. Mahlstedt, C. Maiani, C. Maidantchik, A. A. Maier, T. Maier, A. Maio, S. Majewski, Y. Makida, N. Makovec, B. Malaescu, Pa. Malecki, V. P. Maleev, F. Malek, U. Mallik, D. Malon, C. Malone, S. Maltezos, S. Malyukov, J. Mamuzic, G. Mancini, B. Mandelli, L. Mandelli, I. Mandić, J. Maneira, L. Manhaes de Andrade Filho, J. Manjarres Ramos, A. Mann, A. Manousos, B. Mansoulie, J. D. Mansour, R. Mantifel, M. Mantoani, S. Manzoni, L. Mapelli, G. Marceca, L. March, G. Marchiori, M. Marcisovsky, M. Marjanovic, D. E. Marley, F. Marroquim, S. P. Marsden, Z. Marshall, S. Marti-Garcia, B. Martin, T. A. Martin, V. J. Martin, B. Martin dit Latour, M. Martinez, S. Martin-Haugh, V. S. Martoiu, A. C. Martyniuk, M. Marx, A. Marzin, L. Masetti, T. Mashimo, R. Mashinistov, J. Masik, A. L. Maslennikov, I. Massa, L. Massa, P. Mastrandrea, A. Mastroberardino, T. Masubuchi, P. Mättig, J. Mattmann, J. Maurer, S. J. Maxfield, D. A. Maximov, R. Mazini, S. M. Mazza, N. C. Mc Fadden, G. Mc Goldrick, S. P. Mc Kee, A. McCarn, R. L. McCarthy, T. G. McCarthy, L. I. McClymont, E. F. McDonald, K. W. McFarlane, J. A. Mcfayden, G. Mchedlidze, S. J. McMahon, R. A. McPherson, M. Medinnis, S. Meehan, S. Mehlhase, A. Mehta, K. Meier, C. Meineck, B. Meirose, D. Melini, B. R. Mellado Garcia, M. Melo, F. Meloni, A. Mengarelli, S. Menke, E. Meoni, S. Mergelmeyer, P. Mermod, L. Merola, C. Meroni, F. S. Merritt, A. Messina, J. Metcalfe, A. S. Mete, C. Meyer, C. Meyer, J.-P. Meyer, J. Meyer, H. Meyer Zu Theenhausen, F. Miano, R. P. Middleton, S. Miglioranzi, L. Mijović, G. Mikenberg, M. Mikestikova, M. Mikuž, M. Milesi, A. Milic, D. W. Miller, C. Mills, A. Milov, D. A. Milstead, A. A. Minaenko, Y. Minami, I. A. Minashvili, A. I. Mincer, B. Mindur, M. Mineev, Y. Ming, L. M. Mir, K. P. Mistry, T. Mitani, J. Mitrevski, V. A. Mitsou, A. Miucci, P. S. Miyagawa, J. U. Mjörnmark, T. Moa, K. Mochizuki, S. Mohapatra, S. Molander, R. Moles-Valls, R. Monden, M. C. Mondragon, K. Mönig, J. Monk, E. Monnier, A. Montalbano, J. Montejo Berlingen, F. Monticelli, S. Monzani, R. W. Moore, N. Morange, D. Moreno, M. Moreno Llácer, P. Morettini, D. Mori, T. Mori, M. Morii, M. Morinaga, V. Morisbak, S. Moritz, A. K. Morley, G. Mornacchi, J. D. Morris, S. S. Mortensen, L. Morvaj, M. Mosidze, J. Moss, K. Motohashi, R. Mount, E. Mountricha, S. V. Mouraviev, E. J. W. Moyse, S. Muanza, R. D. Mudd, F. Mueller, J. Mueller, R. S. P. Mueller, T. Mueller, D. Muenstermann, P. Mullen, G. A. Mullier, F. J. Munoz Sanchez, J. A. Murillo Quijada, W. J. Murray, H. Musheghyan, M. Muškinja, A. G. Myagkov, M. Myska, B. P. Nachman, O. Nackenhorst, K. Nagai, R. Nagai, K. Nagano, Y. Nagasaka, K. Nagata, M. Nagel, E. Nagy, A. M. Nairz, Y. Nakahama, K. Nakamura, T. Nakamura, I. Nakano, H. Namasivayam, R. F. Naranjo Garcia, R. Narayan, D. I. Narrias Villar, I. Naryshkin, T. Naumann, G. Navarro, R. Nayyar, H. A. Neal, P. Yu. Nechaeva, T. J. Neep, P. D. Nef, A. Negri, M. Negrini, S. Nektarijevic, C. Nellist, A. Nelson, S. Nemecek, P. Nemethy, A. A. Nepomuceno, M. Nessi, M. S. Neubauer, M. Neumann, R. M. Neves, P. Nevski, P. R. Newman, D. H. Nguyen, T. Nguyen Manh, R. B. Nickerson, R. Nicolaidou, J. Nielsen, A. Nikiforov, V. Nikolaenko, I. Nikolic-Audit, K. Nikolopoulos, J. K. Nilsen, P. Nilsson, Y. Ninomiya, A. Nisati, R. Nisius, T. Nobe, L. Nodulman, M. Nomachi, I. Nomidis, T. Nooney, S. Norberg, M. Nordberg, N. Norjoharuddeen, O. Novgorodova, S. Nowak, M. Nozaki, L. Nozka, K. Ntekas, E. Nurse, F. Nuti, F. O’grady, D. C. O’Neil, A. A. O’Rourke, V. O’Shea, F. G. Oakham, H. Oberlack, T. Obermann, J. Ocariz, A. Ochi, I. Ochoa, J. P. Ochoa-Ricoux, S. Oda, S. Odaka, H. Ogren, A. Oh, S. H. Oh, C. C. Ohm, H. Ohman, H. Oide, H. Okawa, Y. Okumura, T. Okuyama, A. Olariu, L. F. Oleiro Seabra, S. A. Olivares Pino, D. Oliveira Damazio, A. Olszewski, J. Olszowska, A. Onofre, K. Onogi, P. U. E. Onyisi, M. J. Oreglia, Y. Oren, D. Orestano, N. Orlando, R. S. Orr, B. Osculati, R. Ospanov, G. Otero y Garzon, H. Otono, M. Ouchrif, F. Ould-Saada, A. Ouraou, K. P. Oussoren, Q. Ouyang, M. Owen, R. E. Owen, V. E. Ozcan, N. Ozturk, K. Pachal, A. Pacheco Pages, L. Pacheco Rodriguez, C. Padilla Aranda, M. Pagáčová, S. Pagan Griso, F. Paige, P. Pais, K. Pajchel, G. Palacino, S. Palestini, M. Palka, D. Pallin, A. Palma, E. St. Panagiotopoulou, C. E. Pandini, J. G. Panduro Vazquez, P. Pani, S. Panitkin, D. Pantea, L. Paolozzi, Th. D. Papadopoulou, K. Papageorgiou, A. Paramonov, D. Paredes Hernandez, A. J. Parker, M. A. Parker, K. A. Parker, F. Parodi, J. A. Parsons, U. Parzefall, V. R. Pascuzzi, E. Pasqualucci, S. Passaggio, Fr. Pastore, G. Pásztor, S. Pataraia, J. R. Pater, T. Pauly, J. Pearce, B. Pearson, L. E. Pedersen, M. Pedersen, S. Pedraza Lopez, R. Pedro, S. V. Peleganchuk, D. Pelikan, O. Penc, C. Peng, H. Peng, J. Penwell, B. S. Peralva, M. M. Perego, D. V. Perepelitsa, E. Perez Codina, L. Perini, H. Pernegger, S. Perrella, R. Peschke, V. D. Peshekhonov, K. Peters, R. F. Y. Peters, B. A. Petersen, T. C. Petersen, E. Petit, A. Petridis, C. Petridou, P. Petroff, E. Petrolo, M. Petrov, F. Petrucci, N. E. Pettersson, A. Peyaud, R. Pezoa, P. W. Phillips, G. Piacquadio, E. Pianori, A. Picazio, E. Piccaro, M. Piccinini, M. A. Pickering, R. Piegaia, J. E. Pilcher, A. D. Pilkington, A. W. J. Pin, M. Pinamonti, J. L. Pinfold, A. Pingel, S. Pires, H. Pirumov, M. Pitt, L. Plazak, M.-A. Pleier, V. Pleskot, E. Plotnikova, P. Plucinski, D. Pluth, R. Poettgen, L. Poggioli, D. Pohl, G. Polesello, A. Poley, A. Policicchio, R. Polifka, A. Polini, C. S. Pollard, V. Polychronakos, K. Pommès, L. Pontecorvo, B. G. Pope, G. A. Popeneciu, D. S. Popovic, A. Poppleton, S. Pospisil, K. Potamianos, I. N. Potrap, C. J. Potter, C. T. Potter, G. Poulard, J. Poveda, V. Pozdnyakov, M. E. Pozo Astigarraga, P. Pralavorio, A. Pranko, S. Prell, D. Price, L. E. Price, M. Primavera, S. Prince, M. Proissl, K. Prokofiev, F. Prokoshin, S. Protopopescu, J. Proudfoot, M. Przybycien, D. Puddu, M. Purohit, P. Puzo, J. Qian, G. Qin, Y. Qin, A. Quadt, W. B. Quayle, M. Queitsch-Maitland, D. Quilty, S. Raddum, V. Radeka, V. Radescu, S. K. Radhakrishnan, P. Radloff, P. Rados, F. Ragusa, G. Rahal, J. A. Raine, S. Rajagopalan, M. Rammensee, C. Rangel-Smith, M. G. Ratti, F. Rauscher, S. Rave, T. Ravenscroft, I. Ravinovich, M. Raymond, A. L. Read, N. P. Readioff, M. Reale, D. M. Rebuzzi, A. Redelbach, G. Redlinger, R. Reece, K. Reeves, L. Rehnisch, J. Reichert, H. Reisin, C. Rembser, H. Ren, M. Rescigno, S. Resconi, O. L. Rezanova, P. Reznicek, R. Rezvani, R. Richter, S. Richter, E. Richter-Was, O. Ricken, M. Ridel, P. Rieck, C. J. Riegel, J. Rieger, O. Rifki, M. Rijssenbeek, A. Rimoldi, M. Rimoldi, L. Rinaldi, B. Ristić, E. Ritsch, I. Riu, F. Rizatdinova, E. Rizvi, C. Rizzi, S. H. Robertson, A. Robichaud-Veronneau, D. Robinson, J. E. M. Robinson, A. Robson, C. Roda, Y. Rodina, A. Rodriguez Perez, D. Rodriguez Rodriguez, S. Roe, C. S. Rogan, O. Røhne, A. Romaniouk, M. Romano, S. M. Romano Saez, E. Romero Adam, N. Rompotis, M. Ronzani, L. Roos, E. Ros, S. Rosati, K. Rosbach, P. Rose, O. Rosenthal, N.-A. Rosien, V. Rossetti, E. Rossi, L. P. Rossi, J. H. N. Rosten, R. Rosten, M. Rotaru, I. Roth, J. Rothberg, D. Rousseau, C. R. Royon, A. Rozanov, Y. Rozen, X. Ruan, F. Rubbo, M. S. Rudolph, F. Rühr, A. Ruiz-Martinez, Z. Rurikova, N. A. Rusakovich, A. Ruschke, H. L. Russell, J. P. Rutherfoord, N. Ruthmann, Y. F. Ryabov, M. Rybar, G. Rybkin, S. Ryu, A. Ryzhov, G. F. Rzehorz, A. F. Saavedra, G. Sabato, S. Sacerdoti, H. F.-W. Sadrozinski, R. Sadykov, F. Safai Tehrani, P. Saha, M. Sahinsoy, M. Saimpert, T. Saito, H. Sakamoto, Y. Sakurai, G. Salamanna, A. Salamon, J. E. Salazar Loyola, D. Salek, P. H. Sales De Bruin, D. Salihagic, A. Salnikov, J. Salt, D. Salvatore, F. Salvatore, A. Salvucci, A. Salzburger, D. Sammel, D. Sampsonidis, A. Sanchez, J. Sánchez, V. Sanchez Martinez, H. Sandaker, R. L. Sandbach, H. G. Sander, M. Sandhoff, C. Sandoval, R. Sandstroem, D. P. C. Sankey, M. Sannino, A. Sansoni, C. Santoni, R. Santonico, H. Santos, I. Santoyo Castillo, K. Sapp, A. Sapronov, J. G. Saraiva, B. Sarrazin, O. Sasaki, Y. Sasaki, K. Sato, G. Sauvage, E. Sauvan, G. Savage, P. Savard, C. Sawyer, L. Sawyer, J. Saxon, C. Sbarra, A. Sbrizzi, T. Scanlon, D. A. Scannicchio, M. Scarcella, V. Scarfone, J. Schaarschmidt, P. Schacht, B. M. Schachtner, D. Schaefer, R. Schaefer, J. Schaeffer, S. Schaepe, S. Schaetzel, U. Schäfer, A. C. Schaffer, D. Schaile, R. D. Schamberger, V. Scharf, V. A. Schegelsky, D. Scheirich, M. Schernau, C. Schiavi, S. Schier, C. Schillo, M. Schioppa, S. Schlenker, K. R. Schmidt-Sommerfeld, K. Schmieden, C. Schmitt, S. Schmitt, S. Schmitz, B. Schneider, U. Schnoor, L. Schoeffel, A. Schoening, B. D. Schoenrock, E. Schopf, M. Schott, J. Schovancova, S. Schramm, M. Schreyer, N. Schuh, M. J. Schultens, H.-C. Schultz-Coulon, H. Schulz, M. Schumacher, B. A. Schumm, Ph. Schune, A. Schwartzman, T. A. Schwarz, Ph. Schwegler, H. Schweiger, Ph. Schwemling, R. Schwienhorst, J. Schwindling, T. Schwindt, G. Sciolla, F. Scuri, F. Scutti, J. Searcy, P. Seema, S. C. Seidel, A. Seiden, F. Seifert, J. M. Seixas, G. Sekhniaidze, K. Sekhon, S. J. Sekula, D. M. Seliverstov, N. Semprini-Cesari, C. Serfon, L. Serin, L. Serkin, M. Sessa, R. Seuster, H. Severini, T. Sfiligoj, F. Sforza, A. Sfyrla, E. Shabalina, N. W. Shaikh, L. Y. Shan, R. Shang, J. T. Shank, M. Shapiro, P. B. Shatalov, K. Shaw, S. M. Shaw, A. Shcherbakova, C. Y. Shehu, P. Sherwood, L. Shi, S. Shimizu, C. O. Shimmin, M. Shimojima, M. Shiyakova, A. Shmeleva, D. Shoaleh Saadi, M. J. Shochet, S. Shojaii, S. Shrestha, E. Shulga, M. A. Shupe, P. Sicho, A. M. Sickles, P. E. Sidebo, O. Sidiropoulou, D. Sidorov, A. Sidoti, F. Siegert, Dj. Sijacki, J. Silva, S. B. Silverstein, V. Simak, O. Simard, Lj. Simic, S. Simion, E. Simioni, B. Simmons, D. Simon, M. Simon, P. Sinervo, N. B. Sinev, M. Sioli, G. Siragusa, S. Yu. Sivoklokov, J. Sjölin, T. B. Sjursen, M. B. Skinner, H. P. Skottowe, P. Skubic, M. Slater, T. Slavicek, M. Slawinska, K. Sliwa, R. Slovak, V. Smakhtin, B. H. Smart, L. Smestad, J. Smiesko, S. Yu. Smirnov, Y. Smirnov, L. N. Smirnova, O. Smirnova, M. N. K. Smith, R. W. Smith, M. Smizanska, K. Smolek, A. A. Snesarev, S. Snyder, R. Sobie, F. Socher, A. Soffer, D. A. Soh, G. Sokhrannyi, C. A. Solans Sanchez, M. Solar, E. Yu. Soldatov, U. Soldevila, A. A. Solodkov, A. Soloshenko, O. V. Solovyanov, V. Solovyev, P. Sommer, H. Son, H. Y. Song, A. Sood, A. Sopczak, V. Sopko, V. Sorin, D. Sosa, C. L. Sotiropoulou, R. Soualah, A. M. Soukharev, D. South, B. C. Sowden, S. Spagnolo, M. Spalla, M. Spangenberg, F. Spanò, D. Sperlich, F. Spettel, R. Spighi, G. Spigo, L. A. Spiller, M. Spousta, R. D. St. Denis, A. Stabile, R. Stamen, S. Stamm, E. Stanecka, R. W. Stanek, C. Stanescu, M. Stanescu-Bellu, M. M. Stanitzki, S. Stapnes, E. A. Starchenko, G. H. Stark, J. Stark, P. Staroba, P. Starovoitov, S. Stärz, R. Staszewski, P. Steinberg, B. Stelzer, H. J. Stelzer, O. Stelzer-Chilton, H. Stenzel, G. A. Stewart, J. A. Stillings, M. C. Stockton, M. Stoebe, G. Stoicea, P. Stolte, S. Stonjek, A. R. Stradling, A. Straessner, M. E. Stramaglia, J. Strandberg, S. Strandberg, A. Strandlie, M. Strauss, P. Strizenec, R. Ströhmer, D. M. Strom, R. Stroynowski, A. Strubig, S. A. Stucci, B. Stugu, N. A. Styles, D. Su, J. Su, R. Subramaniam, S. Suchek, Y. Sugaya, M. Suk, V. V. Sulin, S. Sultansoy, T. Sumida, S. Sun, X. Sun, J. E. Sundermann, K. Suruliz, G. Susinno, M. R. Sutton, S. Suzuki, M. Svatos, M. Swiatlowski, I. Sykora, T. Sykora, D. Ta, C. Taccini, K. Tackmann, J. Taenzer, A. Taffard, R. Tafirout, N. Taiblum, H. Takai, R. Takashima, T. Takeshita, Y. Takubo, M. Talby, A. A. Talyshev, K. G. Tan, J. Tanaka, R. Tanaka, S. Tanaka, B. B. Tannenwald, S. Tapia Araya, S. Tapprogge, S. Tarem, G. F. Tartarelli, P. Tas, M. Tasevsky, T. Tashiro, E. Tassi, A. Tavares Delgado, Y. Tayalati, A. C. Taylor, G. N. Taylor, P. T. E. Taylor, W. Taylor, F. A. Teischinger, P. Teixeira-Dias, K. K. Temming, D. Temple, H. Ten Kate, P. K. Teng, J. J. Teoh, F. Tepel, S. Terada, K. Terashi, J. Terron, S. Terzo, M. Testa, R. J. Teuscher, T. Theveneaux-Pelzer, J. P. Thomas, J. Thomas-Wilsker, E. N. Thompson, P. D. Thompson, A. S. Thompson, L. A. Thomsen, E. Thomson, M. Thomson, M. J. Tibbetts, R. E. Ticse Torres, V. O. Tikhomirov, Yu. A. Tikhonov, S. Timoshenko, P. Tipton, S. Tisserant, K. Todome, T. Todorov, S. Todorova-Nova, J. Tojo, S. Tokár, K. Tokushuku, E. Tolley, L. Tomlinson, M. Tomoto, L. Tompkins, K. Toms, B. Tong, E. Torrence, H. Torres, E. Torró Pastor, J. Toth, F. Touchard, D. R. Tovey, T. Trefzger, A. Tricoli, I. M. Trigger, S. Trincaz-Duvoid, M. F. Tripiana, W. Trischuk, B. Trocmé, A. Trofymov, C. Troncon, M. Trottier-McDonald, M. Trovatelli, L. Truong, M. Trzebinski, A. Trzupek, J. C.-L. Tseng, P. V. Tsiareshka, G. Tsipolitis, N. Tsirintanis, S. Tsiskaridze, V. Tsiskaridze, E. G. Tskhadadze, K. M. Tsui, I. I. Tsukerman, V. Tsulaia, S. Tsuno, D. Tsybychev, A. Tudorache, V. Tudorache, A. N. Tuna, S. A. Tupputi, S. Turchikhin, D. Turecek, D. Turgeman, R. Turra, A. J. Turvey, P. M. Tuts, M. Tyndel, G. Ucchielli, I. Ueda, R. Ueno, M. Ughetto, F. Ukegawa, G. Unal, A. Undrus, G. Unel, F. C. Ungaro, Y. Unno, C. Unverdorben, J. Urban, P. Urquijo, P. Urrejola, G. Usai, A. Usanova, L. Vacavant, V. Vacek, B. Vachon, C. Valderanis, E. Valdes Santurio, N. Valencic, S. Valentinetti, A. Valero, L. Valery, S. Valkar, S. Vallecorsa, J. A. Valls Ferrer, W. Van Den Wollenberg, P. C. Van Der Deijl, R. van der Geer, H. van der Graaf, N. van Eldik, P. van Gemmeren, J. Van Nieuwkoop, I. van Vulpen, M. C. van Woerden, M. Vanadia, W. Vandelli, R. Vanguri, A. Vaniachine, P. Vankov, G. Vardanyan, R. Vari, E. W. Varnes, T. Varol, D. Varouchas, A. Vartapetian, K. E. Varvell, J. G. Vasquez, F. Vazeille, T. Vazquez Schroeder, J. Veatch, L. M. Veloce, F. Veloso, S. Veneziano, A. Ventura, M. Venturi, N. Venturi, A. Venturini, V. Vercesi, M. Verducci, W. Verkerke, J. C. Vermeulen, A. Vest, M. C. Vetterli, O. Viazlo, I. Vichou, T. Vickey, O. E. Vickey Boeriu, G. H. A. Viehhauser, S. Viel, L. Vigani, R. Vigne, M. Villa, M. Villaplana Perez, E. Vilucchi, M. G. Vincter, V. B. Vinogradov, C. Vittori, I. Vivarelli, S. Vlachos, M. Vlasak, M. Vogel, P. Vokac, G. Volpi, M. Volpi, H. von der Schmitt, E. von Toerne, V. Vorobel, K. Vorobev, M. Vos, R. Voss, J. H. Vossebeld, N. Vranjes, M. Vranjes Milosavljevic, V. Vrba, M. Vreeswijk, R. Vuillermet, I. Vukotic, Z. Vykydal, P. Wagner, W. Wagner, H. Wahlberg, S. Wahrmund, J. Wakabayashi, J. Walder, R. Walker, W. Walkowiak, V. Wallangen, C. Wang, C. Wang, F. Wang, H. Wang, H. Wang, J. Wang, J. Wang, K. Wang, R. Wang, S. M. Wang, T. Wang, T. Wang, W. Wang, X. Wang, C. Wanotayaroj, A. Warburton, C. P. Ward, D. R. Wardrope, A. Washbrook, P. M. Watkins, A. T. Watson, M. F. Watson, G. Watts, S. Watts, B. M. Waugh, S. Webb, M. S. Weber, S. W. Weber, J. S. Webster, A. R. Weidberg, B. Weinert, J. Weingarten, C. Weiser, H. Weits, P. S. Wells, T. Wenaus, T. Wengler, S. Wenig, N. Wermes, M. Werner, M. D. Werner, P. Werner, M. Wessels, J. Wetter, K. Whalen, N. L. Whallon, A. M. Wharton, A. White, M. J. White, R. White, D. Whiteson, F. J. Wickens, W. Wiedenmann, M. Wielers, P. Wienemann, C. Wiglesworth, L. A. M. Wiik-Fuchs, A. Wildauer, F. Wilk, H. G. Wilkens, H. H. Williams, S. Williams, C. Willis, S. Willocq, J. A. Wilson, I. Wingerter-Seez, F. Winklmeier, O. J. Winston, B. T. Winter, M. Wittgen, J. Wittkowski, S. J. Wollstadt, M. W. Wolter, H. Wolters, B. K. Wosiek, J. Wotschack, M. J. Woudstra, K. W. Wozniak, M. Wu, M. Wu, S. L. Wu, X. Wu, Y. Wu, T. R. Wyatt, B. M. Wynne, S. Xella, D. Xu, L. Xu, B. Yabsley, S. Yacoob, R. Yakabe, D. Yamaguchi, Y. Yamaguchi, A. Yamamoto, S. Yamamoto, T. Yamanaka, K. Yamauchi, Y. Yamazaki, Z. Yan, H. Yang, H. Yang, Y. Yang, Z. Yang, W.-M. Yao, Y. C. Yap, Y. Yasu, E. Yatsenko, K. H. Yau Wong, J. Ye, S. Ye, I. Yeletskikh, A. L. Yen, E. Yildirim, K. Yorita, R. Yoshida, K. Yoshihara, C. Young, C. J. S. Young, S. Youssef, D. R. Yu, J. Yu, J. M. Yu, J. Yu, L. Yuan, S. P. Y. Yuen, I. Yusuff, B. Zabinski, R. Zaidan, A. M. Zaitsev, N. Zakharchuk, J. Zalieckas, A. Zaman, S. Zambito, L. Zanello, D. Zanzi, C. Zeitnitz, M. Zeman, A. Zemla, J. C. Zeng, Q. Zeng, K. Zengel, O. Zenin, T. Ženiš, D. Zerwas, D. Zhang, F. Zhang, G. Zhang, H. Zhang, J. Zhang, L. Zhang, R. Zhang, R. Zhang, X. Zhang, Z. Zhang, X. Zhao, Y. Zhao, Z. Zhao, A. Zhemchugov, J. Zhong, B. Zhou, C. Zhou, L. Zhou, L. Zhou, M. Zhou, N. Zhou, C. G. Zhu, H. Zhu, J. Zhu, Y. Zhu, X. Zhuang, K. Zhukov, A. Zibell, D. Zieminska, N. I. Zimine, C. Zimmermann, S. Zimmermann, Z. Zinonos, M. Zinser, M. Ziolkowski, L. Živković, G. Zobernig, A. Zoccoli, M. zur Nedden, G. Zurzolo, L. Zwalinski, ATLAS Collaboration

**Affiliations:** 1 Department of Physics, University of Adelaide, Adelaide, SA Australia; 2 Physics Department, SUNY Albany, Albany, NY USA; 3 Department of Physics, University of Alberta, Edmonton, AB Canada; 4 Department of Physics, Ankara University, Ankara, Turkey; 5 Istanbul Aydin University, Istanbul, Turkey; 6 Division of Physics, TOBB University of Economics and Technology, Ankara, Turkey; 7 LAPP, CNRS/IN2P3 and Université Savoie Mont Blanc, Annecy-le-Vieux, France; 8 High Energy Physics Division, Argonne National Laboratory, Argonne, IL USA; 9 Department of Physics, University of Arizona, Tucson, AZ USA; 10 Department of Physics, The University of Texas at Arlington, Arlington, TX USA; 11 Physics Department, University of Athens, Athens, Greece; 12 Physics Department, National Technical University of Athens, Zografou, Greece; 13 Department of Physics, The University of Texas at Austin, Austin, TX USA; 14 Institute of Physics, Azerbaijan Academy of Sciences, Baku, Azerbaijan; 15 Institut de Física d’Altes Energies (IFAE) The Barcelona Institute of Science and Technology, Barcelona, Spain; 16 Institute of Physics, University of Belgrade, Belgrade, Serbia; 17 Department for Physics and Technology, University of Bergen, Bergen, Norway; 18 Physics Division, Lawrence Berkeley National Laboratory and University of California, Berkeley, CA USA; 19 Department of Physics, Humboldt University, Berlin, Germany; 20 Albert Einstein Center for Fundamental Physics and Laboratory for High Energy Physics, University of Bern, Bern, Switzerland; 21 School of Physics and Astronomy, University of Birmingham, Birmingham, UK; 22 Department of Physics, Bogazici University, Istanbul, Turkey; 23 Department of Physics Engineering, Gaziantep University, Gaziantep, Turkey; 24 Faculty of Engineering and Natural Sciences, Istanbul Bilgi University, Gaziantep, Turkey; 25 Faculty of Engineering and Natural Sciences, Bahcesehir University, Istanbul, Turkey; 26 Centro de Investigaciones, Universidad Antonio Narino, Bogota, Colombia; 27 INFN Sezione di Bologna, Bologna, Italy; 28 Dipartimento di Fisica e Astronomia, Università di Bologna, Bologna, Italy; 29 Physikalisches Institut, University of Bonn, Bonn, Germany; 30 Department of Physics, Boston University, Boston, MA USA; 31 Department of Physics, Brandeis University, Waltham, MA USA; 32 Universidade Federal do Rio De Janeiro COPPE/EE/IF, Rio de Janeiro, Brazil; 33 Electrical Circuits Department, Federal University of Juiz de Fora (UFJF), Juiz de Fora, Brazil; 34 Federal University of Sao Joao del Rei (UFSJ), Sao Joao del Rei, Brazil; 35 Instituto de Fisica, Universidade de Sao Paulo, São Paulo, Brazil; 36 Physics Department, Brookhaven National Laboratory, Upton, NY USA; 37 Transilvania University of Brasov, Brasov, Romania; 38 National Institute of Physics and Nuclear Engineering, Bucharest, Romania; 39 Physics Department, National Institute for Research and Development of Isotopic and Molecular Technologies, Cluj Napoca, Romania; 40 University Politehnica Bucharest, Bucharest, Romania; 41 West University in Timisoara, Timisoara, Romania; 42 Departamento de Física, Universidad de Buenos Aires, Buenos Aires, Argentina; 43 Cavendish Laboratory, University of Cambridge, Cambridge, UK; 44 Department of Physics, Carleton University, Ottawa, ON Canada; 45 CERN, Geneva, Switzerland; 46 Enrico Fermi Institute, University of Chicago, Chicago, IL USA; 47 Departamento de Física, Pontificia Universidad Católica de Chile, Santiago, Chile; 48 Departamento de Física, Universidad Técnica Federico Santa María, Valparaiso, Chile; 49 Institute of High Energy Physics, Chinese Academy of Sciences, Beijing, China; 50 Department of Modern Physics, University of Science and Technology of China, Hefei, Anhui China; 51 Department of Physics, Nanjing University, Nanjing, Jiangsu China; 52 School of Physics, Shandong University, Jinan, Shandong China; 53 Department of Physics and Astronomy, Shanghai Key Laboratory for Particle Physics and Cosmology, Shanghai Jiao Tong University, Shanghai, China; 54 Physics Department, Tsinghua University, Beijing, 100084 China; 55 Laboratoire de Physique Corpusculaire, Clermont Université and Université Blaise Pascal and CNRS/IN2P3, Clermont-Ferrand, France; 56 Nevis Laboratory, Columbia University, Irvington, NY USA; 57 Niels Bohr Institute, University of Copenhagen, Copenhagen, Denmark; 58 INFN Gruppo Collegato di Cosenza, Laboratori Nazionali di Frascati, Frascati, Italy; 59 Dipartimento di Fisica, Università della Calabria, Rende, Italy; 60 Faculty of Physics and Applied Computer Science, AGH University of Science and Technology, Kraków, Poland; 61 Marian Smoluchowski Institute of Physics, Jagiellonian University, Kraków, Poland; 62 Institute of Nuclear Physics Polish Academy of Sciences, Kraków, Poland; 63 Physics Department, Southern Methodist University, Dallas, TX USA; 64 Physics Department, University of Texas at Dallas, Richardson, TX USA; 65 DESY, Hamburg, Zeuthen, Germany; 66 Lehrstuhl für Experimentelle Physik IV, Technische Universität Dortmund, Dortmund, Germany; 67 Institut für Kern-und Teilchenphysik, Technische Universität Dresden, Dresden, Germany; 68 Department of Physics, Duke University, Durham, NC USA; 69 SUPA-School of Physics and Astronomy, University of Edinburgh, Edinburgh, UK; 70 INFN Laboratori Nazionali di Frascati, Frascati, Italy; 71 Fakultät für Mathematik und Physik, Albert-Ludwigs-Universität, Freiburg, Germany; 72 Section de Physique, Université de Genève, Geneva, Switzerland; 73 INFN Sezione di Genova, Genoa, Italy; 74 Dipartimento di Fisica, Università di Genova, Genoa, Italy; 75 E. Andronikashvili Institute of Physics, Iv. Javakhishvili Tbilisi State University, Tbilisi, Georgia; 76 High Energy Physics Institute, Tbilisi State University, Tbilisi, Georgia; 77 II Physikalisches Institut, Justus-Liebig-Universität Giessen, Giessen, Germany; 78 SUPA-School of Physics and Astronomy, University of Glasgow, Glasgow, UK; 79 II Physikalisches Institut, Georg-August-Universität, Göttingen, Germany; 80 Laboratoire de Physique Subatomique et de Cosmologie, Université Grenoble-Alpes, CNRS/IN2P3, Grenoble, France; 81 Department of Physics, Hampton University, Hampton, VA USA; 82 Laboratory for Particle Physics and Cosmology, Harvard University, Cambridge, MA USA; 83 Kirchhoff-Institut für Physik, Ruprecht-Karls-Universität Heidelberg, Heidelberg, Germany; 84 Physikalisches Institut, Ruprecht-Karls-Universität Heidelberg, Heidelberg, Germany; 85 ZITI Institut für technische Informatik, Ruprecht-Karls-Universität Heidelberg, Mannheim, Germany; 86 Faculty of Applied Information Science, Hiroshima Institute of Technology, Hiroshima, Japan; 87 Department of Physics, The Chinese University of Hong Kong, Shatin, NT Hong Kong; 88 Department of Physics, The University of Hong Kong, Hong Kong, China; 89 Department of Physics, The Hong Kong University of Science and Technology, Clear Water Bay, Kowloon, Hong Kong, China; 90 Department of Physics, Indiana University, Bloomington, IN USA; 91 Institut für Astro- und Teilchenphysik, Leopold-Franzens-Universität, Innsbruck, Austria; 92 University of Iowa, Iowa City, IA USA; 93 Department of Physics and Astronomy, Iowa State University, Ames, IA USA; 94 Joint Institute for Nuclear Research, JINR Dubna, Dubna, Russia; 95 KEK, High Energy Accelerator Research Organization, Tsukuba, Japan; 96 Graduate School of Science, Kobe University, Kobe, Japan; 97 Faculty of Science, Kyoto University, Kyoto, Japan; 98 Kyoto University of Education, Kyoto, Japan; 99 Department of Physics, Kyushu University, Fukuoka, Japan; 100 Instituto de Física La Plata, Universidad Nacional de La Plata and CONICET, La Plata, Argentina; 101 Physics Department, Lancaster University, Lancaster, UK; 102 INFN Sezione di Lecce, Lecce, Italy; 103 Dipartimento di Matematica e Fisica, Università del Salento, Lecce, Italy; 104 Oliver Lodge Laboratory, University of Liverpool, Liverpool, UK; 105 Department of Physics, Jožef Stefan Institute and University of Ljubljana, Ljubljana, Slovenia; 106 School of Physics and Astronomy, Queen Mary University of London, London, UK; 107 Department of Physics, Royal Holloway University of London, Surrey, UK; 108 Department of Physics and Astronomy, University College London, London, UK; 109 Louisiana Tech University, Ruston, LA USA; 110 Laboratoire de Physique Nucléaire et de Hautes Energies, UPMC and Université Paris-Diderot and CNRS/IN2P3, Paris, France; 111 Fysiska institutionen, Lunds universitet, Lund, Sweden; 112 Departamento de Fisica Teorica C-15, Universidad Autonoma de Madrid, Madrid, Spain; 113 Institut für Physik, Universität Mainz, Mainz, Germany; 114 School of Physics and Astronomy, University of Manchester, Manchester, UK; 115 CPPM, Aix-Marseille Université and CNRS/IN2P3, Marseille, France; 116 Department of Physics, University of Massachusetts, Amherst, MA USA; 117 Department of Physics, McGill University, Montreal, QC Canada; 118 School of Physics, University of Melbourne, Melbourne, VIC Australia; 119 Department of Physics, The University of Michigan, Ann Arbor, MI USA; 120 Department of Physics and Astronomy, Michigan State University, East Lansing, MI USA; 121 INFN Sezione di Milano, Milan, Italy; 122 Dipartimento di Fisica, Università di Milano, Milan, Italy; 123 B.I. Stepanov Institute of Physics, National Academy of Sciences of Belarus, Minsk, Republic of Belarus; 124 National Scientific and Educational Centre for Particle and High Energy Physics, Minsk, Republic of Belarus; 125 Group of Particle Physics, University of Montreal, Montreal, QC Canada; 126 P.N. Lebedev Physical Institute of the Russian, Academy of Sciences, Moscow, Russia; 127 Institute for Theoretical and Experimental Physics (ITEP), Moscow, Russia; 128 National Research Nuclear University MEPhI, Moscow, Russia; 129 D.V. Skobeltsyn Institute of Nuclear Physics, M.V. Lomonosov Moscow State University, Moscow, Russia; 130 Fakultät für Physik, Ludwig-Maximilians-Universität München, Munich, Germany; 131 Max-Planck-Institut für Physik (Werner-Heisenberg-Institut), Munich, Germany; 132 Nagasaki Institute of Applied Science, Nagasaki, Japan; 133 Graduate School of Science and Kobayashi-Maskawa Institute, Nagoya University, Nagoya, Japan; 134 INFN Sezione di Napoli, Naples, Italy; 135 Dipartimento di Fisica, Università di Napoli, Naples, Italy; 136 Department of Physics and Astronomy, University of New Mexico, Albuquerque, NM USA; 137 Institute for Mathematics, Astrophysics and Particle Physics, Radboud University Nijmegen/Nikhef, Nijmegen, The Netherlands; 138 Nikhef National Institute for Subatomic Physics and University of Amsterdam, Amsterdam, The Netherlands; 139 Department of Physics, Northern Illinois University, DeKalb, IL USA; 140 Budker Institute of Nuclear Physics, SB RAS, Novosibirsk, Russia; 141 Department of Physics, New York University, New York, NY USA; 142 Ohio State University, Columbus, OH USA; 143 Faculty of Science, Okayama University, Okayama, Japan; 144 Homer L. Dodge Department of Physics and Astronomy, University of Oklahoma, Norman, OK USA; 145 Department of Physics, Oklahoma State University, Stillwater, OK USA; 146 Palacký University, RCPTM, Olomouc, Czech Republic; 147 Center for High Energy Physics, University of Oregon, Eugene, OR USA; 148 LAL, Univ. Paris-Sud, CNRS/IN2P3, Université Paris-Saclay, Orsay, France; 149 Graduate School of Science, Osaka University, Osaka, Japan; 150 Department of Physics, University of Oslo, Oslo, Norway; 151 Department of Physics, Oxford University, Oxford, UK; 152 INFN Sezione di Pavia, Pavia, Italy; 153 Dipartimento di Fisica, Università di Pavia, Pavia, Italy; 154 Department of Physics, University of Pennsylvania, Philadelphia, PA USA; 155 National Research Centre “Kurchatov Institute” B.P.Konstantinov Petersburg Nuclear Physics Institute, St. Petersburg, Russia; 156 INFN Sezione di Pisa, Pisa, Italy; 157 Dipartimento di Fisica E. Fermi, Università di Pisa, Pisa, Italy; 158 Department of Physics and Astronomy, University of Pittsburgh, Pittsburgh, PA USA; 159 Laboratório de Instrumentação e Física Experimental de Partículas-LIP, Lisbon, Portugal; 160 Faculdade de Ciências, Universidade de Lisboa, Lisbon, Portugal; 161 Department of Physics, University of Coimbra, Coimbra, Portugal; 162 Centro de Física Nuclear da Universidade de Lisboa, Lisbon, Portugal; 163 Departamento de Fisica, Universidade do Minho, Braga, Portugal; 164 Departamento de Fisica Teorica y del Cosmos and CAFPE, Universidad de Granada, Granada, Spain; 165 Dep Fisica and CEFITEC of Faculdade de Ciencias e Tecnologia, Universidade Nova de Lisboa, Caparica, Portugal; 166 Institute of Physics, Academy of Sciences of the Czech Republic, Prague, Czech Republic; 167 Czech Technical University in Prague, Prague, Czech Republic; 168 Faculty of Mathematics and Physics, Charles University in Prague, Prague, Czech Republic; 169 State Research Center Institute for High Energy Physics (Protvino), NRC KI, Protvino, Russia; 170 Particle Physics Department, Rutherford Appleton Laboratory, Didcot, UK; 171 INFN Sezione di Roma, Rome, Italy; 172 Dipartimento di Fisica, Sapienza Università di Roma, Rome, Italy; 173 INFN Sezione di Roma Tor Vergata, Rome, Italy; 174 Dipartimento di Fisica, Università di Roma Tor Vergata, Rome, Italy; 175 INFN Sezione di Roma Tre, Rome, Italy; 176 Dipartimento di Matematica e Fisica, Università Roma Tre, Rome, Italy; 177 Faculté des Sciences Ain Chock, Réseau Universitaire de Physique des Hautes Energies-Université Hassan II, Casablanca, Morocco; 178 Centre National de l’Energie des Sciences Techniques Nucleaires, Rabat, Morocco; 179 Faculté des Sciences Semlalia, Université Cadi Ayyad, LPHEA-Marrakech, Marrakech, Morocco; 180 Faculté des Sciences, Université Mohamed Premier and LPTPM, Oujda, Morocco; 181 Faculté des Sciences, Université Mohammed V, Rabat, Morocco; 182 DSM/IRFU (Institut de Recherches sur les Lois Fondamentales de l’Univers), CEA Saclay (Commissariat à l’Energie Atomique et aux Energies Alternatives), Gif-sur-Yvette, France; 183 Santa Cruz Institute for Particle Physics, University of California Santa Cruz, Santa Cruz, CA USA; 184 Department of Physics, University of Washington, Seattle, WA USA; 185 Department of Physics and Astronomy, University of Sheffield, Sheffield, UK; 186 Department of Physics, Shinshu University, Nagano, Japan; 187 Fachbereich Physik, Universität Siegen, Siegen, Germany; 188 Department of Physics, Simon Fraser University, Burnaby, BC Canada; 189 SLAC National Accelerator Laboratory, Stanford, CA USA; 190 Faculty of Mathematics, Physics and Informatics, Comenius University, Bratislava, Slovak Republic; 191 Department of Subnuclear Physics, Institute of Experimental Physics of the Slovak Academy of Sciences, Kosice, Slovak Republic; 192 Department of Physics, University of Cape Town, Cape Town, South Africa; 193 Department of Physics, University of Johannesburg, Johannesburg, South Africa; 194 School of Physics, University of the Witwatersrand, Johannesburg, South Africa; 195 Department of Physics, Stockholm University, Stockholm, Sweden; 196 The Oskar Klein Centre, Stockholm, Sweden; 197 Physics Department, Royal Institute of Technology, Stockholm, Sweden; 198 Departments of Physics and Astronomy and Chemistry, Stony Brook University, Stony Brook, NY USA; 199 Department of Physics and Astronomy, University of Sussex, Brighton, UK; 200 School of Physics, University of Sydney, Sydney, NSW Australia; 201 Institute of Physics, Academia Sinica, Taipei, Taiwan; 202 Department of Physics, Technion: Israel Institute of Technology, Haifa, Israel; 203 Raymond and Beverly Sackler School of Physics and Astronomy, Tel Aviv University, Tel Aviv, Israel; 204 Department of Physics, Aristotle University of Thessaloniki, Thessaloniki, Greece; 205 International Center for Elementary Particle Physics and Department of Physics, The University of Tokyo, Tokyo, Japan; 206 Graduate School of Science and Technology, Tokyo Metropolitan University, Tokyo, Japan; 207 Department of Physics, Tokyo Institute of Technology, Tokyo, Japan; 208 Department of Physics, University of Toronto, Toronto, ON Canada; 209 TRIUMF, Vancouver, BC Canada; 210 Department of Physics and Astronomy, York University, Toronto, ON Canada; 211 Faculty of Pure and Applied Sciences and Center for Integrated Research in Fundamental Science and Engineering, University of Tsukuba, Tsukuba, Japan; 212 Department of Physics and Astronomy, Tufts University, Medford, MA USA; 213 Department of Physics and Astronomy, University of California Irvine, Irvine, CA USA; 214 INFN Gruppo Collegato di Udine, Sezione di Trieste, Udine, Italy; 215 ICTP, Trieste, Italy; 216 Dipartimento di Chimica Fisica e Ambiente, Università di Udine, Udine, Italy; 217 Department of Physics and Astronomy, University of Uppsala, Uppsala, Sweden; 218 Department of Physics, University of Illinois, Urbana, IL USA; 219 Instituto de Fisica Corpuscular (IFIC) and Departamento de Fisica Atomica, Molecular y Nuclear and Departamento de Ingeniería Electrónica and Instituto de Microelectrónica de Barcelona (IMB-CNM), University of Valencia and CSIC, Valencia, Spain; 220 Department of Physics, University of British Columbia, Vancouver, BC Canada; 221 Department of Physics and Astronomy, University of Victoria, Victoria, BC Canada; 222 Department of Physics, University of Warwick, Coventry, UK; 223 Waseda University, Tokyo, Japan; 224 Department of Particle Physics, The Weizmann Institute of Science, Rehovot, Israel; 225 Department of Physics, University of Wisconsin, Madison, WI USA; 226 Fakultät für Physik und Astronomie, Julius-Maximilians-Universität, Würzburg, Germany; 227 Fakultät für Mathematik und Naturwissenschaften, Fachgruppe Physik, Bergische Universität Wuppertal, Wuppertal, Germany; 228 Department of Physics, Yale University, New Haven, CT USA; 229 Yerevan Physics Institute, Yerevan, Armenia; 230 Centre de Calcul de l’Institut National de Physique Nucléaire et de Physique des Particules (IN2P3), Villeurbanne, France; 231 CERN, 1211 Geneva 23, Switzerland

## Abstract

The algorithms used by the ATLAS Collaboration to reconstruct and identify prompt photons are described. Measurements of the photon identification efficiencies are reported, using 4.9 fb$^{-1}$ of *pp* collision data collected at the LHC at $\sqrt{s} = 7$ $\text {TeV}$ and 20.3 fb$^{-1}$ at $\sqrt{s} = 8$ $\text {TeV}$. The efficiencies are measured separately for converted and unconverted photons, in four different pseudorapidity regions, for transverse momenta between 10 $\text {GeV}$ and 1.5 $\text {TeV}$. The results from the combination of three data-driven techniques are compared to the predictions from a simulation of the detector response, after correcting the electromagnetic shower momenta in the simulation for the average differences observed with respect to data. Data-to-simulation efficiency ratios used as correction factors in physics measurements are determined to account for the small residual efficiency differences. These factors are measured with uncertainties between 0.5% and 10% in 7 $\text {TeV}$ data and between 0.5% and 5.6% in 8 $\text {TeV}$ data, depending on the photon transverse momentum and pseudorapidity.

## Introduction

Several physics processes occurring in proton–proton collisions at the Large Hadron Collider (LHC) produce final states with *prompt photons*, i.e. photons not originating from hadron decays. The main contributions come from non-resonant production of photons in association with jets or of photon pairs, with cross sections respectively of the order of tens of nanobarns or picobarns [1–6]. The study of such final states, and the measurement of their production cross sections, are of great interest as they probe the perturbative regime of QCD and can provide useful information about the parton distribution functions of the proton [7]. Prompt photons are also produced in rarer processes that are key to the LHC physics programme, such as diphoton decays of the Higgs boson discovered with a mass near 125 $\text {GeV}$, produced with a cross section times branching ratio of about 20 fb at $\sqrt{s}=8$ $\text {TeV}$ [8]. Finally, some expected signatures of physics beyond the Standard Model (SM) are characterised by the presence of prompt photons in the final state. These include resonant photon pairs from graviton decays in models with extra spatial dimensions [9], pairs of photons accompanied by large missing transverse momentum produced in the decays of pairs of supersymmetric particles [10] and events with highly energetic photons and jets from decays of excited quarks or other exotic scenarios [11].

The identification of prompt photons in hadronic collisions is particularly challenging since an overwhelming majority of reconstructed photons is due to *background photons*. These are usually real photons originating from hadron decays in processes with larger cross sections than prompt-photon production. An additional smaller component of background photon candidates is due to hadrons producing in the detector energy deposits that have characteristics similar to those of real photons.

Prompt photons are separated from background photons in the ATLAS experiment by means of selections on quantities describing the shape and properties of the associated electromagnetic showers and by requiring them to be isolated from other particles in the event. An estimate of the efficiency of the photon identification criteria can be obtained from Monte Carlo (MC) simulation. Such an estimate, however, is subject to large, ${\mathcal {O}}(10\%)$, systematic uncertainties. These uncertainties arise from limited knowledge of the detector material, from an imperfect description of the shower development and from the detector response [1]. Ultimately, for high-precision measurements and for accurate comparisons with the predictions from the SM or from theories beyond the SM, a determination of the photon identification efficiency with an uncertainty of ${\mathcal {O}}(1\%)$ or smaller is needed in a large energy range from 10 $\text {GeV}$ to several $\text {TeV}$. This can only be achieved through the use of data control samples. However, this can present several difficulties since there is no single physics process that produces a pure sample of prompt photons in a large transverse momentum ($E_{\text {T}}$) range.

In this document, the reconstruction and identification of photons by the ATLAS detector are described, as well as the measurements of the identification efficiency. This study considers both photons that do (called *converted photons* in the following) or do not convert (called *unconverted photons* in the following) to electron–positron pairs in the detector material upstream of the ATLAS electromagnetic calorimeter. The measurements use the full Run-1 *pp* collision dataset recorded at centre-of-mass energies of 7 and 8 $\text {TeV}$. The details of the selections and the results are given for the data collected in 2012 at $\sqrt{s}=8$ $\text {TeV}$. The same algorithms are applied with minor differences to the $\sqrt{s}=7$ $\text {TeV}$ data collected in 2011.

To overcome the difficulties arising from the absence of a single, pure control sample of prompt photons over a large $E_{\text {T}}$  range, three different data-driven techniques are used. A first method selects photons from radiative decays of the $Z$ boson, i.e. $Z\rightarrow \ell \ell \gamma$ (*Radiative* $Z$ method). A second one extrapolates photon properties from electrons and positrons from *Z* boson decays by exploiting the similarity of the photon and electron interactions with the ATLAS electromagnetic calorimeter (*Electron Extrapolation* method). A third approach exploits a technique to determine the fraction of background present in a sample of isolated photon candidates (*Matrix Method*). Each of these techniques can measure the photon identification efficiency in complementary but overlapping $E_{\text {T}}$ regions with varying precision.

This document is organised as follows. After an overview of the ATLAS detector in Sect. 2, the photon reconstruction and identification algorithms used in ATLAS are detailed in Sect. 3. Section 4 summarises the data and simulation samples used and describes the corrections applied to the simulated photon shower shapes in order to improve agreement with the data. In Sect. 5 the three data-driven approaches to the measurement of the photon identification efficiency are described, listing their respective sources of uncertainty and the precision reached in the relevant $E_{\text {T}}$ ranges. The results obtained with the $\sqrt{s}=8$ TeV data collected in 2012, their consistency in the overlapping $E_{\text {T}}$ intervals and the comparison to the MC predictions are presented in Sect. 6. Results obtained for the identification criteria used during the 2011 data-taking period at $\sqrt{s}=7$ $\text {TeV}$ are described in Sect. 7. Finally, Sect. 8 discusses the impact of multiple inelastic interactions in the same beam crossing on the photon identification efficiency.

## ATLAS detector

The ATLAS experiment [12] is a multi-purpose particle detector with approximately forward-backward symmetric cylindrical geometry and nearly 4$\pi$ coverage in solid angle.^1^

The inner tracking detector (ID), surrounded by a thin superconducting solenoid providing a $2\,{\mathrm {T}}$ axial magnetic field, provides precise reconstruction of tracks within a pseudorapidity range $|\eta | \lesssim 2.5$. The innermost part of the ID consists of a silicon pixel detector $(50.5~\hbox {mm}<r<150~\hbox {mm})$ providing typically three measurement points for charged particles originating in the beam-interaction region. The layer closest to the beam pipe (referred to as the *b*-layer in this paper) contributes significantly to precision vertexing and provides discrimination between prompt tracks and photon conversions. A semiconductor tracker (SCT) consisting of modules with two layers of silicon microstrip sensors surrounds the pixel detector, providing typically eight hits per track at intermediate radii $(275~\hbox {mm}< r < 560~\hbox {mm}).$ The outermost region of the ID $(563~\hbox {mm}< r < 1066~\hbox {mm})$ is covered by a transition radiation tracker (TRT) consisting of straw drift tubes filled with a xenon gas mixture, interleaved with polypropylene/polyethylene transition radiators. For charged particles with transverse momentum $p_{\text {T}} >0.5$ $\text {GeV}$ within its pseudorapidity coverage ($|\eta | \lesssim 2$), the TRT provides typically 35 hits per track. The distinction between transition radiation (low-energy photons emitted by electrons traversing the radiators) and tracking signals is obtained on a straw-by-straw basis using separate low and high thresholds in the front-end electronics. The inner detector allows an accurate reconstruction and transverse momentum measurement of tracks from the primary proton–proton collision region. It also identifies tracks from secondary vertices, permitting the efficient reconstruction of photon conversions up to a radial distance of about 80 cm from the beamline.

**Fig. 1:**
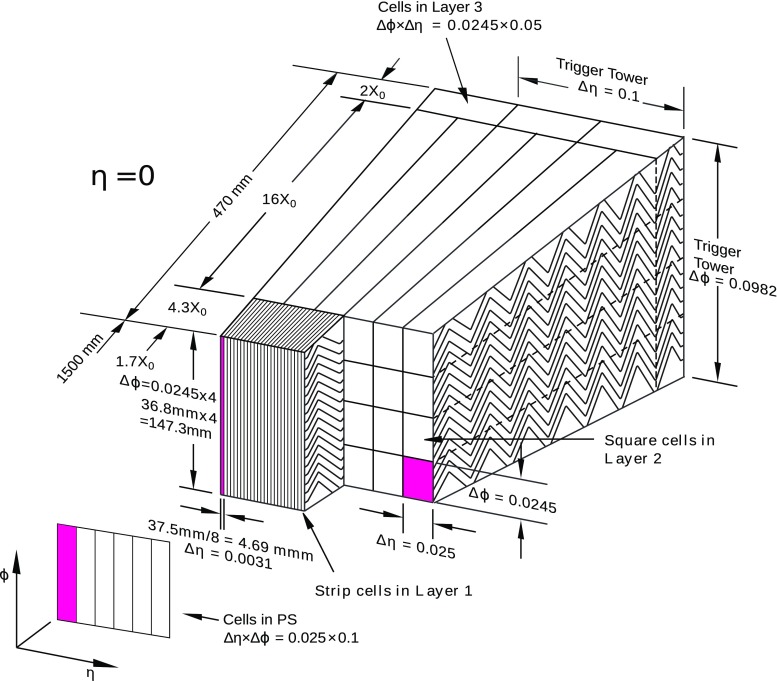
Sketch of a barrel module (located at $\eta =0$) of the ATLAS electromagnetic calorimeter. The different longitudinal layers (one presampler, PS, and three layers in the accordion calorimeter) are depicted. The granularity in $\eta$ and $\phi$ of the cells of each layer and of the trigger towers is also shown

The solenoid is surrounded by a high-granularity lead/liquid-argon (LAr) sampling electromagnetic (EM) calorimeter with an accordion geometry. The EM calorimeter measures the energy and the position of electromagnetic showers with $|\eta |<3.2$. It is divided into a barrel section, covering the pseudorapidity region $|\eta | < 1.475$, and two end-cap sections, covering the pseudorapidity regions $1.375< |\eta | < 3.2$. The transition region between the barrel and the end-caps, $1.37< |\eta | < 1.52$, has a large amount of material upstream of the first active calorimeter layer. The EM calorimeter is composed, for $|\eta |<2.5$, of three sampling layers, longitudinal in shower depth. The first layer has a thickness of about 4.4 radiation lengths ($X_0$). In the ranges $|\eta |<1.4$ and $1.5< |\eta | <2.4$, the first layer is segmented into high-granularity strips in the $\eta$ direction, with a typical cell size of $0.003\times 0.0982$ in $\Delta \eta \times \Delta \phi$ in the barrel. For $1.4<|\eta |<1.5$ and $2.4< |\eta | <2.5$ the $\eta$-segmentation of the first layer is coarser, and the cell size is $\Delta \eta \times \Delta \phi = 0.025\times 0.0982$. The fine $\eta$ granularity of the strips is sufficient to provide, for transverse momenta up to ${\mathcal {O}}(100~\mathrm {GeV})$, an event-by-event discrimination between single photon showers and two overlapping showers coming from the decays of neutral hadrons, mostly $\pi ^0$ and $\eta$ mesons, in jets in the fiducial pseudorapidity region $|\eta |<1.37$ or $1.52<|\eta |<2.37$. The second layer has a thickness of about 17 $X_0$ and a granularity of $0.025 \times 0.0245$ in $\Delta \eta \times \Delta \phi$. It collects most of the energy deposited in the calorimeter by photon and electron showers. The third layer has a granularity of $0.05\times 0.0245$ in $\Delta \eta \times \Delta \phi$ and a depth of about 2 $X_0$. It is used to correct for leakage beyond the EM calorimeter of high-energy showers. In front of the accordion calorimeter, a thin presampler layer, covering the pseudorapidity interval $|\eta | < 1.8$, is used to correct for energy loss upstream of the calorimeter. The presampler consists of an active LAr layer with a thickness of 1.1 cm (0.5 cm) in the barrel (end-cap) and has a granularity of $\Delta \eta \times \Delta \phi = 0.025 \times 0.0982$. The material upstream of the presampler has a thickness of about 2 $X_0$ for $|\eta |<0.6$. In the region $0.6<|\eta |<0.8$ this thickness increases linearly from 2 $X_0$ to 3 $X_0$. For $0.8<|\eta |<1.8$ the material thickness is about or slightly larger than 3 $X_0$, with the exception of the transition region between the barrel and the end-caps and the region near $|\eta |=1.7$, where it reaches 5–6 $X_0$. A sketch of a barrel module of the electromagnetic calorimeter is shown in Fig. 1.

The hadronic calorimeter surrounds the EM calorimeter. It consists of an iron–scintillator tile calorimeter in the central region ($|\eta |< 1.7$), and LAr sampling calorimeters with copper and tungsten absorbers in the end-cap ($1.5<|\eta |<3.2$) and forward ($3.1<|\eta |<4.9$) regions.

The muon spectrometer surrounds the calorimeters. It consists of three large superconducting air-core toroid magnets, each with eight coils, a system of precision tracking chambers ($|\eta |<2.7$), and fast tracking chambers ($|\eta |<2.4$) for triggering.

A three-level trigger system selects events to be recorded for offline analysis. A coarser readout granularity (corresponding to the “towers” of Fig. 1) is used by the first-level trigger, while the full detector granularity is exploited by the higher-level trigger. To reduce the data acquisition rate of low-threshold triggers, used for collecting various control samples, prescale factors (*N*) can be applied to each trigger, such that only 1 in *N* events passing the trigger causes an event to be accepted at that trigger level.

## Photon reconstruction and identification

### Photon reconstruction

The electromagnetic shower, originating from an energetic photon’s interaction with the EM calorimeter, deposits a significant amount of energy in a small number of neighbouring calorimeter cells. As photons and electrons have very similar signatures in the EM calorimeter, their reconstruction proceeds in parallel. The electron reconstruction, including a dedicated, cluster-seeded track-finding algorithm to increase the efficiency for the reconstruction of low-momentum electron tracks, is described in Ref. [13]. The reconstruction of unconverted and converted photons proceeds in the following way:

- seed clusters of EM calorimeter cells are searched for;
- tracks reconstructed in the inner detector are loosely matched to seed clusters;
- tracks consistent with originating from a photon conversion are used to create conversion vertex candidates;
- conversion vertex candidates are matched to seed clusters;
- a final algorithm decides whether a seed cluster corresponds to an unconverted photon, a converted photon or a single electron based on the matching to conversion vertices or tracks and on the cluster and track(s) four-momenta.

In the following the various steps of the reconstruction algorithms are described in more detail.

The reconstruction of photon candidates in the region $|\eta |<2.5$ begins with the creation of a preliminary set of seed clusters of EM calorimeter cells. Seed clusters of size $\Delta \eta \times \Delta \phi = 0.075 \times 0.123$ with transverse momentum above 2.5 $\text {GeV}$ are formed by a sliding-window algorithm [14]. After an energy comparison, duplicate clusters of lower energy are removed from nearby seed clusters. From MC simulations, the efficiency of the initial cluster reconstruction is estimated to be greater than 99% for photons with $E_{\text {T}} > 20$ $\text {GeV}$.

Once seed clusters are reconstructed, a search is performed for inner detector tracks [15, 16] that are loosely matched to the clusters, in order to identify and reconstruct electrons and photon conversions. Tracks are loosely matched to a cluster if the angular distance between the cluster barycentre and the extrapolated track’s intersection point with the second sampling layer of the calorimeter is smaller than 0.05 (0.2) along $\phi$ in the direction of (opposite to) the bending of the tracks in the magnetic field of the ATLAS solenoid, and smaller than 0.05 along $\eta$ for tracks with hits in the silicon detectors, i.e. the pixel and SCT detectors. Tracks with hits in the silicon detectors are extrapolated from the point of closest approach to the primary vertex, while tracks without hits in the silicon detectors are extrapolated from the last measured point. The track is extrapolated to the position corresponding to the expected maximum energy deposit for EM showers. To efficiently select low-momentum tracks that may have suffered significant bremsstrahlung losses before reaching the calorimeter, a similar matching procedure is applied after rescaling the track momentum to the measured cluster energy. The previous matching requirements are applied except that the $\phi$ difference in the direction of bending should be smaller than 0.1. Tracks that are loosely matched to a cluster and with hits in the silicon detectors are refitted with a Gaussian-sum-filter technique [17, 18], to improve the track parameter resolution, and are retained for the reconstruction of electrons and converted photons.

“Double-track” conversion vertex candidates are reconstructed from pairs of oppositely charged tracks in the ID that are likely to be electrons. For each track the likelihood to be an electron, based on high-threshold hits and time-over-threshold of low-threshold hits in the TRT, is required to be at least 10% (80%) for tracks with (without) hits in the silicon detectors. Since the tracks of a photon conversion are parallel at the place of conversion, geometric requirements are used to select the track pairs. Track pairs are classified into three categories, whether both tracks (Si–Si), none (TRT–TRT) or only one of them (Si–TRT) have hits in the silicon detectors. Track pairs fulfilling the following requirements are retained:

- $\Delta \cot \theta$ between the two tracks (taken at the tracks’ points of closest approach to the primary vertex) is less than 0.3 for Si–Si track pairs and 0.5 for track pairs with at least one track without hits in the silicon detectors. This requirement is not applied for TRT–TRT track pairs with both tracks within $|\eta |<0.6$.
- The distance of closest approach between the two tracks is less than 10 mm for Si–Si track pairs and 50 mm for track pairs with at least one track without hits in the silicon detectors.
- The difference between the sum of the radii of the helices that can be constructed from the electron and positron tracks and the distance between the centres of the two helices is between $-5$ and 5 mm, between $-50$ and 10 mm, or between $-25$ and 10 mm, for Si–Si, TRT–TRT and Si–TRT track pairs, respectively.
- $\Delta \phi$ between the two tracks (taken at the estimated vertex position before the conversion vertex fit) is less than 0.05 for Si–Si track pairs and 0.2 for tracks pairs with at least one track without hits in the silicon detectors.

A constrained conversion vertex fit with three degrees of freedom is performed using the five measured helix parameters of each of the two participating tracks with the constraint that the tracks are parallel at the vertex. Only the vertices satisfying the following requirements are retained:

- The $\chi ^2$ of the conversion vertex fit is less than 50. This loose requirement suppresses fake candidates from random combination of tracks while being highly efficient for true photon conversions.
- The radius of the conversion vertex, defined as the distance from the vertex to the beamline in the transverse plane, is greater than 20 mm, 50 mm or 250 mm for vertices from Si–Si, Si–TRT and TRT–TRT track pairs, respectively.
- The difference in $\phi$ between the vertex position and the direction of the reconstructed conversion is less than 0.2.

The efficiency to reconstruct photon conversions as double-track vertex candidates decreases significantly for conversions taking place in the outermost layers of the ID. This effect is due to photon conversions in which one of the two produced electron tracks is not reconstructed either because it is very soft (asymmetric conversions where one of the two tracks has $p_{\text {T}} < 0.5$ $\text {GeV}$), or because the two tracks are very close to each other and cannot be adequately separated. For this reason, tracks without hits in the *b*-layer that either have an electron likelihood greater than 95%, or have no hits in the TRT, are considered as “single-track” conversion vertex candidates. In this case, since a conversion vertex fit cannot be performed, the conversion vertex is defined to be the location of the first measurement of the track. Tracks which pass through a passive region of the *b*-layer are not considered as single-track conversions unless they are missing a hit in the second pixel layer.

As in the loose track matching, the matching of the conversion vertices to the clusters relies on an extrapolation of the conversion candidates to the second sampling layer of the calorimeter, and the comparison of the extrapolated $\eta$ and $\phi$ coordinates to the $\eta$ and $\phi$ coordinates of the cluster centre. The details of the extrapolation depend on the type of the conversion vertex candidate.

- For double-track conversion vertex candidates for which the track transverse momenta differ by less than a factor of four from each other, each track is extrapolated to the second sampling layer of the calorimeter and is required to be matched to the cluster.
- For double-track conversion vertex candidates for which the track transverse momenta differ by more than a factor of four from each other, the photon direction is reconstructed from the electron and positron directions determined by the conversion vertex fit, and used to perform a straight-line extrapolation to the second sampling layer of the calorimeter, as expected for a neutral particle.
- For single-track conversion vertex candidates, the track is extrapolated from its last measurement.

Conversion vertex candidates built from tracks with hits in the silicon detectors are considered matched to a cluster if the angular distance between the extrapolated conversion vertex candidate and the cluster centre is smaller than 0.05 in both $\eta$ and $\phi$. If the extrapolation is performed for single-track conversions, the window in $\phi$ is increased to 0.1 in the direction of the bending. For tracks without hits in the silicon detectors, the matching requirements are tighter:

- The distance in $\phi$ between the extrapolated track(s) and the cluster is less than 0.02 (0.03) in the direction of (opposite to) the bending. In the case where the conversion vertex candidate is extrapolated as a neutral particle, the distance is required to be less than 0.03 on both sides.
- The distance in $\eta$ between the extrapolated track(s) and the cluster is less than 0.35 and 0.2 in the barrel and end-cap sections of the TRT, respectively. The criteria are significantly looser than in the $\phi$ direction since the TRT does not provide a measurement of the pseudorapidity in its barrel section. In the case that the conversion vertex candidate is extrapolated as a neutral particle, the distance is required to be less than 0.35.

In the case of multiple conversion vertex candidates matched to the same cluster, the final conversion vertex candidate is chosen as follows:

- preference is given to double-track candidates over single-track candidates;
- if both conversion vertex candidates are formed from the same number of tracks, preference is given to the candidate with more tracks with hits in the silicon detectors;
- if the conversion vertex candidates are formed from the same number of tracks with hits in the silicon detectors, preference is given to the vertex candidate with smaller radius.

The final arbitration between the unconverted photon, converted photon and electron hypotheses for the reconstructed EM clusters is performed in the following way [19]:

- Clusters to which neither a conversion vertex candidate nor any track has been matched during the electron reconstruction are considered unconverted photon candidates.
- Electromagnetic clusters matched to a conversion vertex candidate are considered converted photon candidates. For converted photon candidates that are also reconstructed as electrons, the electron track is evaluated against the track(s) originating from the conversion vertex candidate matched to the same cluster:
  1. If the track coincides with a track coming from the conversion vertex, the converted photon candidate is retained.
  2. The only exception to the previous rule is the case of a double-track conversion vertex candidate where the coinciding track has a hit in the *b*-layer, while the other track lacks one (for this purpose, a missing hit in a disabled *b*-layer module is counted as a hit^2^).
  3. If the track does not coincide with any of the tracks assigned to the conversion vertex candidate, the converted photon candidate is removed, unless the track $p_{\text {T}}$ is smaller than the $p_{\text {T}}$ of the converted photon candidate.
- Single-track converted photon candidates are recovered from objects that are only reconstructed as electron candidates with $p_{\text {T}} > 2$ $\text {GeV}$ and $E/p < 10$ (*E* being the cluster energy and *p* the track momentum), if the track has no hits in the silicon detectors.
- Unconverted photon candidates are recovered from reconstructed electron candidates if the electron candidate has a corresponding track without hits in the silicon detectors and with $p_{\text {T}} < 2$ $\text {GeV}$, or if the electron candidate is not considered as single-track converted photon and its matched track has a transverse momentum lower than 2 $\text {GeV}$ or *E* / *p* greater than 10. The corresponding electron candidate is then removed from the event. Using this procedure around 85% of the unconverted photons erroneously categorised as electrons are recovered.

From MC simulations, 96% of prompt photons with $E_{\text {T}} >25$ $\text {GeV}$ are expected to be reconstructed as photon candidates, while the remaining 4% are incorrectly reconstructed as electrons but not as photons. The reconstruction efficiencies of photons with transverse momenta of a few tens of $\text {GeV}$ (relevant for the search for Higgs boson decays to two photons) are checked in data with a technique described in Ref. [20]. The results point to inefficiencies and fake rates that exceed by up to a few percent the predictions from MC simulation. The efficiency to reconstruct photon conversions decreases at high $E_{\text {T}}$ (${>}150$ GeV), where it becomes more difficult to separate the two tracks from the conversions. Such conversions with very close-by tracks are often not recovered as single-track conversions because of the tighter selections, including the transition radiation requirement, applied to single-track conversion candidates. The overall photon reconstruction efficiency is thus reduced to about 90% for $E_{\text {T}}$ around 1 $\text {TeV}$.

The final photon energy measurement is performed using information from the calorimeter, with a cluster size that depends on the photon classification.^3^ In the barrel, a cluster of size $\Delta \eta \times \Delta \phi =0.075\times 0.123$ is used for unconverted photon candidates, while a cluster of size $0.075 \times 0.172$ is used for converted photon candidates to compensate for the opening between the conversion products in the $\phi$ direction due to the magnetic field of the ATLAS solenoid. In the end-cap, a cluster size of $0.125\times 0.123$ is used for all candidates. The photon energy calibration, which accounts for upstream energy loss and both lateral and longitudinal leakage, is based on the same procedure that is used for electrons [20, 21] but with different calibration factors for converted and unconverted photon candidates. In the following the photon transverse momentum $E_{\text {T}}$ is computed from the photon cluster’s calibrated energy *E* and the pseudorapidity $\eta _2$ of the barycentre of the cluster in the second layer of the EM calorimeter as $E_{\text {T}} =E/\cosh (\eta _2)$.

### Photon identification

To distinguish prompt photons from background photons, photon identification with high signal efficiency and high background rejection is required for transverse momenta from 10 $\text {GeV}$ to the $\text {TeV}$ scale. Photon identification in ATLAS is based on a set of cuts on several discriminating variables. Such variables, listed in Table 1 and described in Appendix A, characterise the lateral and longitudinal shower development in the electromagnetic calorimeter and the shower leakage fraction in the hadronic calorimeter. Prompt photons typically produce narrower energy deposits in the electromagnetic calorimeter and have smaller leakage to the hadronic one compared to background photons from jets, due to the presence of additional hadrons near the photon candidate in the latter case. In addition, background candidates from isolated $\pi ^0\rightarrow \gamma \gamma$ decays – unlike prompt photons – are often characterised by two separate local energy maxima in the finely segmented strips of the first layer, due to the small separation between the two photons. The distributions of the discriminating variables for both the prompt and background photons are affected by additional soft *pp* interactions that may accompany the hard-scattering collision, referred to as in-time pile-up, as well as by out-of-time pile-up arising from bunches before or after the bunch where the event of interest was triggered. Pile-up results in the presence of low-$E_{\text {T}}$ activity in the detector, including energy deposition in the electromagnetic calorimeter. This effect tends to broaden the distributions of the discriminating variables and thus to reduce the separation between prompt and background photon candidates.

Two reference selections, a *loose* one and a *tight* one, are defined. The *loose* selection is based only on shower shapes in the second layer of the electromagnetic calorimeter and on the energy deposited in the hadronic calorimeter, and is used by the photon triggers. The loose requirements are designed to provide a high prompt-photon identification efficiency with respect to reconstruction. Their efficiency rises from 97% at $E_{\text {T}} ^\gamma = 20~\text {GeV}$ to above 99% for $E_{\text {T}} ^\gamma > 40~\text {GeV}$ for both the converted and unconverted photons, and the corresponding background rejection factor is about 1000 [19]. The rejection factor is defined as the ratio of the number of initial jets with $p_{\text {T}} >40$ $\text {GeV}$ in the acceptance of the calorimeter to the number of reconstructed background photon candidates satisfying the identification criteria. The *tight* selection adds information from the finely segmented strip layer of the calorimeter, which provides good rejection of hadronic jets where a neutral meson carries most of the jet energy. The *tight* criteria are separately optimised for unconverted and converted photons to provide a photon identification efficiency of about 85% for photon candidates with transverse energy $E_{\text {T}} > 40~\text {GeV}$ and a corresponding background rejection factor of about 5000 [19].

The selection criteria are different in seven intervals of the reconstructed photon pseudorapidity (0.0–0.6, 0.6–0.8, 0.8–1.15, 1.15–1.37, 1.52–1.81, 1.81–2.01, 2.01–2.37) to account for the calorimeter geometry and for different effects on the shower shapes from the material upstream of the calorimeter, which is highly non-uniform as a function of $|\eta |$.

The photon identification criteria were first optimised prior to the start of the data-taking in 2010, on simulated samples of prompt photons from $\gamma +$jet, diphoton and $H\rightarrow \gamma \gamma$ events and samples of background photons in QCD multi-jet events [19]. Before the 2011 data-taking, the *loose* and the *tight* selections were loosened, without further re-optimisation, in order to reduce the systematic effects associated to the differences between the calorimetric variables measured from data and their description by the ATLAS simulation. Prior to the 8 $\text {TeV}$ run in 2012, the identification criteria were reoptimised based on improved simulations in which the values of the shower shape variables are slightly shifted to improve the agreement with the data shower shapes, as described in the next section. To cope with the higher pile-up expected during the 2012 data taking, the criteria on the shower shapes more sensitive to pile-up were relaxed while the others were tightened.

The discriminating variables that are most sensitive to pile-up are found to be the energy leakage in the hadronic calorimeter and the shower width in the second sampling layer of the EM calorimeter.

**Table 1:** Discriminating variables used for *loose* and *tight* photon identification

Category	Description	Name	*Loose*	*Tight*
Acceptance	$|\eta |<2.37$, with $1.37<|\eta |<1.52$ excluded	–	$\checkmark$	$\checkmark$
Hadronic leakage	Ratio of $E_{\text {T}}$ in the first sampling layer of the hadronic calorimeter to $E_{\text {T}}$ of the EM cluster (used over the range $|\eta | < 0.8$ or $|\eta | > 1.37$)	$R_\mathrm {had_{1}}$	$\checkmark$	$\checkmark$
	Ratio of $E_{\text {T}}$ in the hadronic calorimeter to $E_{\text {T}}$ of the EM cluster (used over the range $0.8< |\eta | < 1.37$)	$R_\mathrm {had}$	$\checkmark$	$\checkmark$
EM middle layer	Ratio of 3 $\times$ 7 $\eta \times \phi$ to 7 $\times$ 7 cell energies	$R_{\eta }$	$\checkmark$	$\checkmark$
	Lateral width of the shower	$w_{\eta _2}$	$\checkmark$	$\checkmark$
	Ratio of 3$\times$3 $\eta \times \phi$ to 3$\times$7 cell energies	$R_{\phi }$		$\checkmark$
EM strip layer	Shower width calculated from three strips around the strip with maximum energy deposit	$w_{s\,3}$		$\checkmark$
	Total lateral shower width	$w_{s\,\mathrm {tot}}$		$\checkmark$
	Energy outside the core of the three central strips but within seven strips divided by energy within the three central strips	$F_{\mathrm {side}}$		$\checkmark$
	Difference between the energy associated with the second maximum in the strip layer and the energy reconstructed in the strip with the minimum value found between the first and second maxima	$\Delta {}E$		$\checkmark$
	Ratio of the energy difference associated with the largest and second largest energy deposits to the sum of these energies	$E_{\mathrm {ratio}}$		$\checkmark$

### Photon isolation

The identification efficiencies presented in this article are measured for photon candidates passing an isolation requirement, similar to those applied to reduce hadronic background in cross-section measurements or searches for exotic processes with photons [1–6, 8, 9, 11, 22]. In the data taken at $\sqrt{s}=8$ $\text {TeV}$, the calorimeter isolation transverse energy $E_{\mathrm {T}}^{\mathrm {iso}}$ [23] is required to be lower than 4 $\text {GeV}$. This quantity is computed from positive-energy three-dimensional topological clusters of calorimeter cells [14] reconstructed in a cone of size $\Delta R = \sqrt{(\Delta \eta )^2+(\Delta \phi )^2}=0.4$ around the photon candidate.

The contributions to $E_{\mathrm {T}}^{\mathrm {iso}}$ from the photon itself and from the underlying event and pile-up are subtracted. The correction for the photon energy outside the cluster is computed as the product of the photon transverse energy and a coefficient determined from separate simulations of converted and unconverted photons. The underlying event and pile-up energy correction is computed on an event-by-event basis using the method described in Refs. [24, 25]. A $k_{\mathrm {T}}$ jet-finding algorithm [26, 27] of size parameter $R =0.5$ is used to reconstruct all jets without any explicit transverse momentum threshold, starting from the three-dimensional topological clusters reconstructed in the calorimeter. Each jet is assigned an area $A_{\mathrm {jet}}$ via a Voronoi tessellation [28] of the $\eta$–$\phi$ space. According to this algorithm, every point within a jet’s assigned area is closer to the axis of that jet than to the axis of any other jet. The ambient transverse energy density $\rho _{\mathrm {UE}}(\eta )$ from pile-up and the underlying event is taken to be the median of the transverse energy densities $p_{\text {T}} ^{\mathrm {jet}}/A_{\mathrm {jet}}$ of jets with pseudorapidity $|\eta |<1.5$ or $1.5<|\eta |<3.0$. The area of the photon isolation cone is then multiplied by $\rho _{\mathrm {UE}}$ to compute the correction to $E_{\mathrm {T}}^{\mathrm {iso}}$. The estimated ambient transverse energy fluctuates significantly event-by-event, reflecting the fluctuations in the underlying event and pile-up activity in the data. The typical size of the correction is 2 $\text {GeV}$ in the central region and 1.5 $\text {GeV}$ in the forward region.

A slight dependence of the identification efficiency on the isolation requirement is observed, as discussed in Sect. 6.2.

## Data and Monte Carlo samples

The data used in this study consist of the 7 and 8 $\text {TeV}$ proton–proton collisions recorded by the ATLAS detector during 2011 and 2012 in LHC Run 1. They correspond respectively to 4.9 fb$^{-1}$ and 20.3 fb$^{-1}$ of integrated luminosity after requiring good data quality. The mean number of interactions per bunch crossing, $\mu$, was 9 and 21 on average in the $\sqrt{s}=7$ and 8 $\text {TeV}$ datasets, respectively.

The *Z* boson radiative decay and the electron extrapolation methods use data collected with the lowest-threshold lepton triggers with prescale factors equal to one and thus exploit the full luminosity of Run 1. For the data collected in 2012 at $\sqrt{s}=8$ $\text {TeV}$, the transverse momentum thresholds for single-lepton triggers are 25 (24) $\text {GeV}$ for $\ell =e~(\mu )$, while those for dilepton triggers are 12 (13) $\text {GeV}$. For the data collected in 2011 at $\sqrt{s}=7$ $\text {TeV}$, the transverse momentum thresholds for single-lepton triggers are 20 (18) $\text {GeV}$ for $\ell =e~(\mu )$, while those for dilepton triggers are 12 (10) $\text {GeV}$. The matrix method uses events collected with single-photon triggers with loose identification requirements and large prescale factors, and thus exploits only a fraction of the total luminosity. Photons reconstructed near regions of the calorimeter affected by read-out or high-voltage failures [29] are rejected.

Monte Carlo samples are processed through a full simulation of the ATLAS detector response [30] using Geant4 [31] 4.9.4-patch04. Pile-up *pp* interactions in the same and nearby bunch crossings are included in the simulation. The MC samples are reweighted to reproduce the distribution of $\mu$ and the length of the luminous region observed in data (approximately 54 cm and 48 cm in the data taken at $\sqrt{s}=7$ and 8 $\text {TeV}$, respectively). Samples of prompt photons are generated with PYTHIA8 [32, 33]. Such samples include the leading-order $\gamma$ + jet events from $q g \rightarrow q \gamma$ and $q \bar{q} \rightarrow g \gamma$ hard scattering, as well as prompt photons from quark fragmentation in QCD dijet events. About $10^7$ events are generated, covering the whole transverse momentum spectrum under study. Samples of background photons in jets are produced by generating with PYTHIA8 all tree-level 2$\rightarrow$2 QCD processes, removing $\gamma$ + jet events from quark fragmentation. Between $1.2\times 10^6$ and $5\times 10^6$ $Z\rightarrow \ell \ell \gamma$ ($\ell =e,\mu$) events are generated with SHERPA [34] or with POWHEG [35, 36] interfaced to PHOTOS [37] for the modelling of QED final-state radiation and to PYTHIA8 for showering, hadronisation and modelling of the underlying event. About $10^7$ $Z(\rightarrow \ell \ell )$+jet events are generated for both $\ell =e$ and $\ell =\mu$ with each of the following three event generators: POWHEG interfaced to PYTHIA8; ALPGEN [38] interfaced to HERWIG [39] and JIMMY [40] for showering, hadronisation and modelling of the underlying event; and SHERPA. A sample of MC $H \rightarrow Z \gamma$ events [41] is also used to compute the efficiency in the simulation for photons with transverse momentum between 10 and 15 $\text {GeV}$, since the $Z\rightarrow \ell \ell \gamma$ samples have a generator-level requirement on the minimum true photon transverse momentum of 10 $\text {GeV}$ which biases the reconstructed transverse momentum near the threshold. A two-dimensional reweighting of the pseudorapidity and transverse momentum spectra of the photons is applied to match the distributions of those reconstructed in $Z\rightarrow \ell \ell \gamma$ events. For the analysis of $\sqrt{s}=7$ TeV data, all simulated samples (photon+jet, QCD multi-jet, $Z(\rightarrow \ell \ell )+$jet and $Z\rightarrow \ell \ell \gamma$) are generated with PYTHIA6.

For the analysis of 8 $\text {TeV}$ data, the events are simulated and reconstructed using the model of the ATLAS detector described in Ref. [20], based on an improved in situ determination of the passive material upstream of the electromagnetic calorimeter. This model is characterised by the presence of additional material (up to 0.6 radiation lengths) in the end-cap and a $50\%$ smaller uncertainty in the material budget with respect to the previous model, which is used for the study of 7 $\text {TeV}$ data.

The distributions of the photon transverse shower shapes in the ATLAS MC simulation do not perfectly match the ones observed in data. While the shapes of these distributions in the simulation are rather similar to those found in the data, small systematic differences in their average values are observed. On the other hand, the longitudinal electromagnetic shower profiles are well described by the simulation. The differences between the data and MC distributions are parameterised as simple shifts and applied to the MC simulated values to match the distributions in data. These shifts are calculated by minimising the $\chi ^2$ between the data and the shifted MC distributions of photon candidates satisfying the *tight* identification criteria and the calorimeter isolation requirement described in the previous section. The shifts are computed in intervals of the reconstructed photon pseudorapidity and transverse momentum. The pseudorapidity intervals are the same as those used to define the photon selection criteria. The $E_{\text {T}}$ bin boundaries are 0, 15, 20, 25, 30, 40, 50, 60, 80, 100 and 1000 $\text {GeV}$. The typical size of the correction factors is 10% of the RMS of the distribution of the corresponding variable in data. For the variable $R_{\eta }$, for which the level of agreement between the data and the simulation is worst, the size of the correction factors is 50% of the RMS of the distribution. The corresponding correction to the prompt-photon efficiency predicted by the simulation varies with pseudorapidity between $-10\%$ and $-5\%$ for photon transverse momenta close to 10 GeV, and approaches zero for transverse momenta above 50 GeV.

**Fig. 2:**
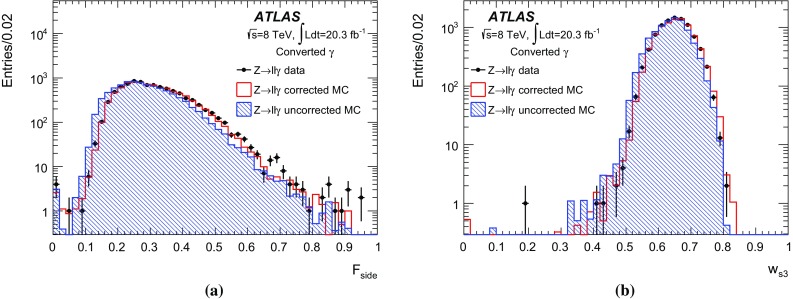
Distributions of the calorimetric discriminating variables **a** $F_{\mathrm {side}}$ and **b** $w_{s\,3}$ for converted photon candidates with $E_{\text {T}} > 20~\text {GeV}$ and $|\eta | <2.37$ (excluding $1.37<|\eta |<1.52$) selected from $Z\rightarrow \ell \ell \gamma$ events obtained from the 2012 data sample (*dots*). The distributions for true photons from simulated $Z\rightarrow \ell \ell \gamma$ events (*blue hatched* and *red hollow histograms*) are also shown, after reweighting their two-dimensional $E_{\text {T}}$ vs $\eta$ distributions to match that of the data candidates. The *blue hatched histogram* corresponds to the uncorrected simulation and the *red hollow* one to the simulation corrected by the average shift between data and simulation distributions determined from the inclusive sample of isolated photon candidates passing the *tight* selection per bin of ($\eta$, $E_{\text {T}}$) and for converted and unconverted photons separately. The photon candidates must be isolated but no shower-shape criteria are applied. The photon purity of the data sample, i.e. the fraction of prompt photons, is estimated to be approximately 99%

Two examples of the simulated discriminating variable distributions before and after corrections, for converted photon candidates originating from *Z* boson radiative decays, are shown in Fig. 2. For comparison, the distributions observed in data for candidates passing the *Z* boson radiative decay selection illustrated in Sect. 5.1, are also shown. Better agreement between the shower shape distributions in data and in the simulation after applying such corrections is clearly visible.

## Techniques to measure the photon identification efficiency

The photon identification efficiency, $\varepsilon _\mathrm {ID}$, is defined as the ratio of the number of isolated photons passing the *tight* identification selection to the total number of isolated photons. Three data-driven techniques are developed in order to measure this efficiency for reconstructed photons over a wide transverse momentum range.

The *Radiative* $Z$ method uses a clean sample of prompt, isolated photons from radiative leptonic decays of the $Z$ boson, $Z\rightarrow \ell \ell \gamma$, in which a photon produced from the final-state radiation of one of the two leptons is selected without imposing any criteria on the photon discriminating variables. Given the luminosity of the data collected in Run 1, this method allows the measurement of the photon identification efficiency only for $10~\text {GeV}\lesssim E_{\text {T}} \lesssim 80$ $\text {GeV}$.

In the *Electron Extrapolation* method, a large and pure sample of electrons selected from $Z\rightarrow ee$ decays with a tag-and-probe technique is used to deduce the distributions of the discriminating variables for photons by exploiting the similarity between the electron and the photon EM showers. Given the typical $E_{\text {T}}$ distribution of electrons from $Z$ boson decays and the Run-1 luminosity, this method provides precise results for $30~\text {GeV}\lesssim E_{\text {T}} \lesssim 100~\text {GeV}$.

The *Matrix Method* uses the discrimination between prompt photons and background photons provided by their isolation from tracks in the ID to extract the sample purity before and after applying the *tight* identification requirements. This method provides results for transverse momenta from 20 $\text {GeV}$ to 1.5 $\text {TeV}$.

The three measurements are performed for photons with pseudorapidity in the fiducial region of the EM calorimeter in which the first layer is finely segmented along $\eta$: $|\eta |<1.37$ or $1.52<|\eta |<2.37$. The identification efficiency is measured as a function of $E_{\text {T}}$ in four pseudorapidity intervals: $|\eta |<0.6$, $0.6<|\eta |<1.37$, $1.52<|\eta |<1.81$ and $1.81<|\eta |<2.37$. Since there are not many data events with high-$E_{\text {T}}$ photons, the highest $E_{\text {T}}$ bin in which the measurement with the matrix method is performed corresponds to the large interval 250 GeV$<E_{\text {T}} <1500$ GeV (the upper limit corresponding to the transverse energy of the highest-$E_{\text {T}}$ selected photon candidate). In this range a majority of the photon candidates have transverse momenta below about 400 GeV (the $E_{\text {T}}$ distribution of the selected photon candidates in this interval has an average value of 300 GeV and an RMS value of 70 GeV). However, from the simulation the photon identification efficiency is expected to be constant at the few per-mil level in this $E_{\text {T}}$ range.

### Photons from $Z$ boson radiative decays

Radiative $Z \rightarrow \ell \ell \gamma$ decays are selected by placing kinematic requirements on the dilepton pair, the invariant mass of the three particles in the final state and quality requirements on the two leptons. The reconstructed photon candidates are required to be isolated in the calorimeter but no selection is applied to their discriminating variables.

Events are collected using the lowest-threshold unprescaled single-lepton or dilepton triggers.

Muon candidates are formed from tracks reconstructed both in the ID and in the muon spectrometer [42], with transverse momentum larger than 15 $\text {GeV}$ and pseudorapidity $|\eta |<2.4$. The muon tracks are required to have at least one hit in the innermost pixel layer, one hit in the other two pixel layers, five hits in the SCT, and at most two missing hits in the two silicon detectors. The muon track isolation, defined as the sum of the transverse momenta of the tracks inside a cone of size $\Delta R=\sqrt{(\Delta \eta )^2 + (\Delta \phi )^2}=0.2$ around the muon, excluding the muon track, is required to be smaller than 10% of the muon $p_{\text {T}}$.

Electron candidates are required to have $E_{\text {T}} > 15$ $\text {GeV}$, and $|\eta |<1.37$ or $1.52<|\eta |<2.47$. Electrons are required to satisfy *medium* identification criteria [43] based on tracking and transition radiation information from the ID, shower shape variables computed from the lateral and longitudinal profiles of the energy deposited in the EM calorimeter, and track–cluster matching quantities.

For both the electron and muon candidates, the longitudinal ($z_{0}$) and transverse ($d_0$) impact parameters of the reconstructed tracks with respect to the primary vertex with at least three associated tracks and with the largest $\sum p_{\mathrm {T}}^2$ of the associated tracks are required to satisfy $|z_0|<10$ mm and $|d_{0}|/\sigma _{d_{0}}<10$, respectively, where $\sigma _{d_{0}}$ is the estimated $d_0$ uncertainty.

The $Z \rightarrow \ell \ell \gamma$ candidates are selected by requiring two opposite-sign charged leptons of the same flavour satisfying the previous criteria and one isolated photon candidate with $E_{\text {T}} >10$ $\text {GeV}$ and $|\eta |<1.37$ or $1.52<|\eta |<2.37$. An angular separation $\Delta R>0.2$ (0.4) between the photon and each of the two muons (electrons) is required so that the energy deposited by the leptons in the calorimeter does not bias the photon discriminating variables. In the selected events, the triggering leptons are required to match one (or in the case of dilepton triggered events, both) of the *Z* candidate’s leptons.

**Fig. 3:**
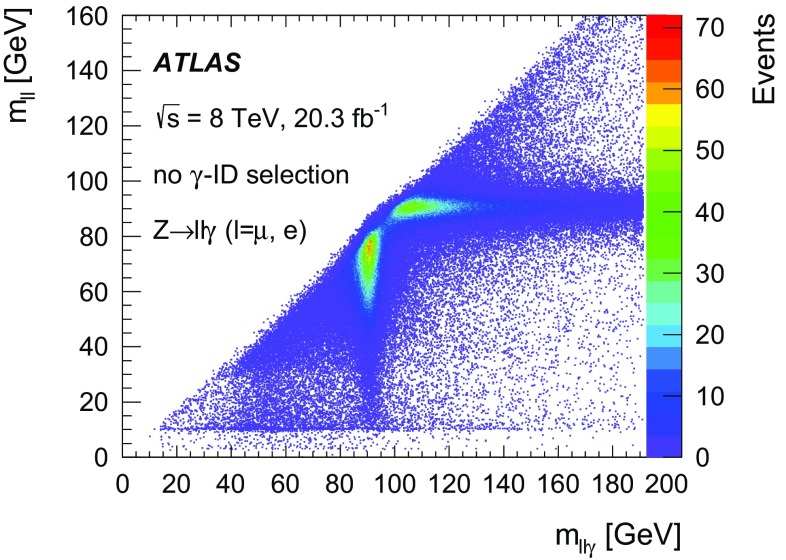
Two-dimensional distribution of $m_{\ell \ell \gamma }$ and $m_{\ell \ell }$ for all reconstructed $Z \rightarrow \ell \ell \gamma$ candidates after loosening the selection applied to $m_{\ell \ell \gamma }$ and $m_{\ell \ell }$. No photon identification requirements are applied. Events from initial-state ($m_{\ell \ell }\approx m_Z$) and final-state ($m_{\ell \ell \gamma }\approx m_Z$) radiation are clearly visible

The two-dimensional distribution of the dilepton invariant mass, $m_{\ell \ell }$, versus the invariant mass of the three final-state particles, $m_{\ell \ell \gamma }$, in events selected in $\sqrt{s}=8$ $\text {TeV}$ data is shown in Fig. 3. The selected sample is dominated by $Z$ +jet background events in which one jet is misreconstructed as a photon. These events, which have a cross section about three orders of magnitudes higher than $\ell \ell \gamma$ events, have $m_{\ell \ell }\approx m_Z$ and $m_{\ell \ell \gamma }\gtrsim m_Z$, while final-state radiation $Z \rightarrow \ell \ell \gamma$ events have $m_{\ell \ell }\lesssim m_Z$ and $m_{\ell \ell \gamma }\approx m_Z$, where $m_Z$ is the $Z$ boson pole mass. To significantly reduce the $Z$ +jet background, the requirements of $40~\text {GeV}<m_{\ell \ell }<83~\text {GeV}$ and $80~\text {GeV}<m_{\ell \ell \gamma }<96~\text {GeV}$ are thus applied.

After the selection, about 54000 unconverted and about 19000 converted isolated photon candidates are selected in the $Z \rightarrow \mu \mu \gamma$ channel, and 32000 unconverted and 12000 converted isolated photon candidates are selected in the $Z \rightarrow ee\gamma$ channel. The residual background contamination from *Z*+jet events is estimated through a maximum-likelihood fit (called “template fit” in the following) to the $m_{\ell \ell \gamma }$ distribution of selected events after discarding the $80~\text {GeV}<m_{\ell \ell \gamma }<96~\text {GeV}$ requirement. The data are fit to a sum of the photon and background contributions. The photon and background $m_{\ell \ell \gamma }$ distributions (“templates”) are extracted from the $Z \rightarrow \ell \ell \gamma$ and $Z$ +jet simulations, corrected to take into account known data–MC differences in the photon and lepton energy scales and resolution and in the lepton efficiencies. The signal and background yields are determined from the data by maximising the likelihood. Due to the small number of selected events in data and simulation, these fits are performed only for two photon transverse momentum intervals, $10~\text {GeV}<E_{\text {T}} <15~\text {GeV}$ and $E_{\text {T}} >15$ $\text {GeV}$, and integrated over the photon pseudorapidity, since the signal purity is found to be similar in the four photon $|\eta |$ intervals within statistical uncertainties.

**Fig. 4:**
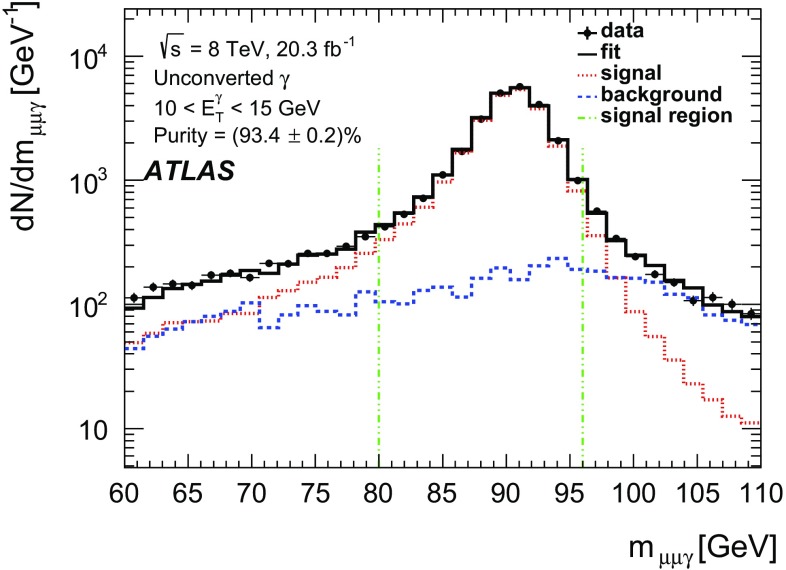
Invariant mass ($m_{\mu \mu \gamma }$) distribution of events in which the unconverted photon has $10~\text {GeV}<E_{\text {T}} <15$ $\text {GeV}$, selected in data at $\sqrt{s}= 8$ $\text {TeV}$ after applying all the $Z \rightarrow \mu \mu \gamma$ selection criteria except that on $m_{\mu \mu \gamma }$ (*black dots*). No photon identification requirements are applied. The *solid black line* represents the result of fitting the data distribution to a sum of the signal (*red dashed line*) and background (*blue dotted line*) invariant mass distributions obtained from simulations

Figure 4 shows the result of the fit for unconverted photon candidates with transverse momenta between 10 GeV and 15 $\text {GeV}$. The fraction of residual background in the region $80~\text {GeV}<m_{\ell \ell \gamma }<96$ $\text {GeV}$ decreases rapidly with the reconstructed photon transverse momentum, from $\approx$10% for $10~\text {GeV}<E_{\text {T}} <15$ $\text {GeV}$ to $\le$ 2% for higher-$E_{\text {T}}$ regions. A similar fit is also performed for the subsample in which the photon candidates are required to satisfy the *tight* identification criteria.

The identification efficiency as a function of $E_{\text {T}}$ is estimated as the fraction of all the selected probes in a certain $E_{\text {T}}$ interval passing the *tight* identification requirements. For $10~\text {GeV}<E_{\text {T}} <15$ $\text {GeV}$, both the numerator and denominator are corrected for the average background fraction determined from the template fit. For $E_{\text {T}} >15$ $\text {GeV}$, the background is neglected in the nominal result, and a systematic uncertainty is assigned as the difference between the nominal result and the efficiency that would be obtained taking into account the background fraction determined from the template fit in this $E_{\text {T}}$ region. Additional systematic uncertainties related to the signal and background $m_{\ell \ell \gamma }$ distributions are estimated by repeating the previous fits with templates extracted from alternative MC event generators (POWHEG interfaced to PHOTOS and PYTHIA8 for $Z\rightarrow \ell \ell \gamma$ and ALPGEN for *Z*+jet, $Z\rightarrow \ell \ell$). The total relative uncertainty in the efficiency, dominated by the statistical component, increases from 1.5–3% (depending on $\eta$ and whether the photon was reconstructed as a converted or an unconverted candidate) for $10~\text {GeV}<E_{\text {T}} <15$ $\text {GeV}$ to 5–20% for $E_{\text {T}} >40$ $\text {GeV}$.

### Electron extrapolation

The similarity between the electromagnetic showers induced by isolated electrons and photons in the EM calorimeter is exploited to extrapolate the expected photon distributions of the discriminating variables. The photon identification efficiency is thus estimated from the distributions of the same variables in a pure and large sample of electrons with $E_{\text {T}}$ between 30 GeV and 100 $\text {GeV}$ obtained from $Z \rightarrow ee$ decays using a tag-and-probe method [43]. Events collected with single-electron triggers are selected if they contain two opposite-sign electrons with $E_{\text {T}} >25$ $\text {GeV}$, $|\eta |<1.37$ or $1.52<|\eta |<2.47$, at least one hit in the pixel detector and seven hits in the silicon detectors, ${E_{\mathrm {T}}^{\mathrm {iso}}}<4$ $\text {GeV}$ and invariant mass $80~\text {GeV}<m_{ee}<120$ $\text {GeV}$. The tag electron is required to match the trigger object and to pass the *tight* electron identification requirements. A sample of about $9\times 10^6$ electron probes is collected. Its purity is determined from the $m_{ee}$ spectrum of the selected events by estimating the background, whose normalisation is extracted using events with $m_{ee}>120$ $\text {GeV}$ and whose shape is obtained from events in which the probe electron candidate fails both the isolation and identification requirements. The purity varies slightly with $E_{\text {T}}$ and $|\eta |$, but is always above 99%.

The differences between the photon and electron distributions of the discriminating variables are studied using simulated samples of prompt photons and electrons from $Z \rightarrow ee$ decays, separately for converted and unconverted photons. The shifts of the photon discriminating variables described in Sect. 4 are not applied, since it is observed that the photon and electron distributions are biased in a similar way in the simulation.

Photon conversions produce electron–positron pairs which are usually sufficiently collimated to produce overlapping showers in the calorimeter, giving rise to single clusters with distributions of the discriminating variables similar to those of an isolated electron. The largest differences between electrons and converted photons are found in the $R_{\phi }$ distribution, due to the bending of electrons and positrons in opposite directions in the *r*–$\phi$ plane, which leads to a broader $R_{\phi }$ distribution for converted photons. However, the $R_{\phi }$ requirement used for the identification of converted photons is relatively loose, and a test on MC simulated samples shows that, by directly applying the converted photon identification criteria to an electron sample, the $\varepsilon _\mathrm {ID}$ obtained from electrons overestimates the efficiency for converted photons by at most 3%.

The showers induced by unconverted photons are more likely to begin later than those induced by electrons, and thus to be narrower in the first layer of the EM calorimeter. Additionally, the lack of photon-trajectory bending in the $\phi$ plane makes the $R_{\phi }$ distribution particularly different from that of electrons. Therefore, if the unconverted-photon selection criteria are directly applied to an electron sample, the $\varepsilon _\mathrm {ID}$ obtained from these electrons is about 20–30% smaller than the efficiency for unconverted photons with the same pseudorapidity and transverse momentum.

**Fig. 5:**
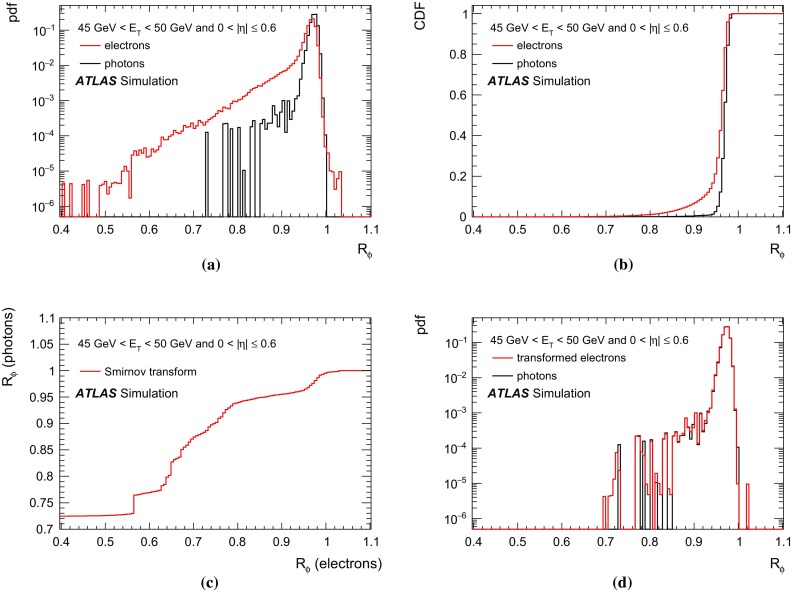
Diagram illustrating the process of Smirnov transformation. $R_{\phi }$ is chosen as an example discriminating variable whose distribution is particularly different between electrons and (unconverted) photons. The $R_{\phi }$ probability density function (pdf) in each sample (**a**) is used to calculate the respective CDF (**b**). From the two CDFs, a Smirnov transformation can be derived (**c**). Applying the transformation leads to an $R_{\phi }$ distribution of the transformed electrons which closely resembles the photon distribution (**d**)

To reduce such effects a mapping technique based on a Smirnov transformation [44] is used for both the unconverted and converted photons. For each discriminating variable *x*, the cumulative distribution functions (CDF) of simulated electrons and photons, $\mathrm {CDF}_e(x)$ and $\mathrm {CDF}_\gamma (x)$, are calculated. The transform *f*(*x*) is thus defined by $\mathrm {CDF}_e(x) = \mathrm {CDF}_\gamma (f(x))$. The discriminating variable of the electron probes selected in data can then be corrected on an event-by-event basis by applying the transform *f*(*x*) to obtain the expected one for photons in data. Figure 5 illustrates the process for one shower shape ($R_{\phi }$). These Smirnov transformations are invariant under systematic shifts which are fully correlated between the electron and photon distributions. Due to the differences in the $|\eta |$ and $E_{\text {T}}$ distributions of the source and target samples, the dependence of the shower shapes on $|\eta |$, $E_{\text {T}}$, and whether the photon was reconstructed as a converted or an unconverted candidate, this process is applied separately for converted and unconverted photons, and in various regions of $E_{\text {T}}$ and $|\eta |$. The efficiency of the identification criteria is determined from the extrapolated photon distributions of the discriminating variables.

The following three sources of systematic uncertainty are considered for this analysis:

- As the Smirnov transformations are obtained independently for each shower shape, the estimated photon identification efficiency can be biased if the correlations among the discriminating variables are significantly different between electrons and photons. Non-closure tests are performed on the simulation, comparing the identification efficiency of true prompt photons with the efficiency extrapolated from electron probes selected with the same requirement as in data and applying the extrapolation procedure. The differences between the true and extrapolated efficiencies are at the level of 1% or less, with a few exceptions for unconverted photons, for which maximum differences of 2% are found.
- The results are also affected by the uncertainties in the modelling of the shower shape distributions and correlations in the photon and electron simulations used to extract the mappings. The largest uncertainties in the distributions of the discriminating variables originate from limited knowledge of the material upstream of the calorimeter. The extraction of the mappings is repeated using alternative MC samples based on a detector simulation with a conservative estimate of additional material in front of the calorimeter [21]. This detector simulation is considered as conservative enough to cover any mismodelling of the distributions of the discriminating variables. The extracted ${\varepsilon _\mathrm {ID}}$ differs from the nominal one by typically less than 1% for converted photons and 2% for unconverted ones, with maximum deviations of 2% and 3.5% in the worst cases, respectively.
- Finally, the effect of a possible background contamination in the selected electron probes in data is found to be smaller than 0.5% in all $E_{\text {T}}$, $|\eta |$ intervals for both the converted and unconverted photons.

The total uncertainty is dominated by its systematic component and ranges from 1.5% in the central region to 7.5% in the highest $E_{\text {T}}$ bin in the endcap region, with typical values of 2.5%.

### Matrix method

An inclusive sample of about $7\times 10^6$ isolated photon candidates is selected using single-photon triggers by requiring at least one photon candidate with transverse momentum 20 $\text {GeV}$ $<E_{\text {T}} <1500$ $\text {GeV}$ and isolation energy ${E_{\mathrm {T}}^{\mathrm {iso}}}<4$ $\text {GeV}$, matched to the photon trigger object passing the *loose* identification requirements.

The distribution of the track isolation of selected candidates in data is used to discriminate between prompt and background photon candidates, before and after applying the *tight* identification criteria. The track isolation variable used for the measurement of the efficiency of unconverted photon candidates, $p_{\mathrm {T}}^{\mathrm {iso}}$, is defined as the scalar sum of the transverse momenta of the tracks, with transverse momentum above 0.5 $\text {GeV}$ and distance of closest approach to the primary vertex along *z* less than 0.5 mm, within a hollow cone of $0.1<\Delta R<0.3$ around the photon direction. For the measurement of the efficiency of the converted photon candidates, the track isolation variable $\nu _{\mathrm {trk}}^{\mathrm {iso}}$ is defined as the number of tracks, passing the previous requirements, within a hollow cone of $0.1<\Delta R<0.4$ around the photon direction. Unconverted photon candidates with $p_{\mathrm {T}}^{\mathrm {iso}}<1.2$ $\text {GeV}$ and converted photon candidates with $\nu _{\mathrm {trk}}^{\mathrm {iso}}=0$ are considered to be isolated from tracks. The track isolation variables and requirements were chosen to minimise the total uncertainty in the identification efficiency after including both the statistical and systematic components.

The yields of prompt and background photons in the selected sample (“ALL” sample), $N^{\mathrm {S}}_{\mathrm {all}}$ and $N^{\mathrm {B}}_{\mathrm {all}}$, and in the sample of candidates satisfying the *tight* identification criteria (“PASS” sample), $N^{\mathrm {S}}_{\mathrm {pass}}$ and $N^{\mathrm {B}}_{\mathrm {pass}}$, are obtained by solving a system of four equations:

1 $$\begin{aligned} N^{\mathrm {T}}_{\mathrm {all}}= & {} N^{\mathrm {S}}_{\mathrm {all}} + N^{\mathrm {B}}_{\mathrm {all}} \nonumber ,\\ N^{\mathrm {T}}_{\mathrm {pass}}= & {} N^{\mathrm {S}}_{\mathrm {pass}} + N^{\mathrm {B}}_{\mathrm {pass}} \nonumber , \\ N^{{\mathrm {T}},{\mathrm {iso}}}_{\mathrm {all}}= & {} \varepsilon ^{\mathrm {S}}_{\mathrm {all}}\times N^{\mathrm {S}}_{\mathrm {all}} + \varepsilon ^{\mathrm {B}}_{\mathrm {all}}\times N^{\mathrm {B}}_{\mathrm {all}} \nonumber ,\\ N^{{\mathrm {T}},{\mathrm {iso}}}_{\mathrm {pass}}= & {} \varepsilon ^{\mathrm {S}}_{\mathrm {pass}} \times N^{\mathrm {S}}_{\mathrm {pass}}+\varepsilon ^{\mathrm {B}}_{\mathrm {pass}} \times N^{\mathrm {B}}_{\mathrm {pass}}. \end{aligned}$$

Here $N^{\mathrm {T}}_{\mathrm {all}}$ and $N^{\mathrm {T}}_{\mathrm {pass}}$ are the total numbers of candidates in the ALL and PASS samples respectively, while $N^{{\mathrm {T}},{\mathrm {iso}}}_{\mathrm {all}}$ and $N^{{\mathrm {T}},{\mathrm {iso}}}_{\mathrm {pass}}$ are the numbers of candidates in the ALL and PASS samples that pass the track isolation requirement. The quantities $\varepsilon _{\mathrm {all}}^{\mathrm {S(B)}}$ and $\varepsilon _{\mathrm {pass}}^{\mathrm {S(B)}}$ are the efficiencies of the track isolation requirements for prompt (background) photons in the ALL and PASS samples.

Equation (1) implies that the fractions $f_{\mathrm {pass}}$ and $f_{\mathrm {all}}$ of prompt photons in the ALL and in the PASS samples can be written as:

2 $$\begin{aligned} f_{\mathrm {pass}}= & {} \frac{\varepsilon _{\mathrm {pass}}-\varepsilon _{\mathrm {pass}}^ {\mathrm {B}}}{\varepsilon _{\mathrm {pass}}^{\mathrm {S}}-\varepsilon _{\mathrm {pass}}^ {\mathrm {B}}}\nonumber \\ f_{\mathrm {all}}= & {} \frac{\varepsilon _{\mathrm {all}}-\varepsilon _{\mathrm {all}}^ {\mathrm {B}}}{\varepsilon _{\mathrm {all}}^{\mathrm {S}}-\varepsilon _{\mathrm {all}}^ {\mathrm {B}}} \end{aligned}$$

where $\varepsilon _{\mathrm {pass(all)}} =N^{{\mathrm {T}},{\mathrm {iso}}}_{\mathrm {pass(all)}}/N^{\mathrm {T}}_{\mathrm {pass(all)}}$ is the fraction of *tight* (all) photon candidates in data that satisfy the track isolation criteria.

The identification efficiency ${\varepsilon _\mathrm {ID}}=N^{\mathrm {S}}_{\mathrm {pass}}/N^ {\mathrm {S}}_{\mathrm {all}}$ is thus:

3 $$\begin{aligned} {\varepsilon _\mathrm {ID}}=\frac{N^{\mathrm {T}}_{\mathrm {pass}}}{N^{\mathrm {T}}_{\mathrm {all}}} \left( \frac{\varepsilon _{\mathrm {pass}}-\varepsilon _{\mathrm {pass}}^{\mathrm {B}}}{\varepsilon _{\mathrm {pass}}^{\mathrm {S}}-\varepsilon _{\mathrm {pass}}^{\mathrm {B}}} \right) \left( \frac{\varepsilon _{\mathrm {all}}-\varepsilon _{\mathrm {all}}^{\mathrm {B}}}{\varepsilon _{\mathrm {all}}^{\mathrm {S}}-\varepsilon _{\mathrm {all}}^{\mathrm {B}}} \right) ^{-1}. \end{aligned}$$

The prompt-photon track isolation efficiencies, $\varepsilon _{\mathrm {all}}^{\mathrm {S}}$ and $\varepsilon _{\mathrm {pass}}^{\mathrm {S}}$, are estimated from simulated prompt-photon events. The difference between the track isolation efficiency for electrons collected in data and simulation with a tag-and-probe $Z \rightarrow ee$ selection is taken as a systematic uncertainty. An additional systematic uncertainty in the prompt-photon track isolation efficiencies is estimated by conservatively varying the fraction of fragmentation photons in the simulation by $\pm 100\%$. The overall uncertainties in $\varepsilon _{\mathrm {all}}^{\mathrm {S}}$ and $\varepsilon _{\mathrm {pass}}^{\mathrm {S}}$ are below 1%.

The background-photon track isolation efficiencies, $\varepsilon ^{\mathrm {B}}_{\mathrm {all}}$ and $\varepsilon ^{\mathrm {B}}_{\mathrm {pass}}$, are estimated from data samples enriched in background photons. For the measurement of $\varepsilon ^{\mathrm {B}}_{\mathrm {all}}$, the control sample of all photon candidates not meeting at least one of the *tight* identification criteria is used. In order to obtain $\varepsilon _{\mathrm {pass}}^{\mathrm {B}}$, a relaxed version of the *tight* identification criteria is defined. The *relaxed tight* selection consists of those candidates which fail at least one of the requirements on four discriminating variables computed from the energy in the cells of the first EM calorimeter layer ($F_{\mathrm {side}}$, $w_{s3}$, $\Delta E$, $E_{\mathrm {ratio}}$), but satisfy the remaining *tight* identification criteria. The four variables which are removed from the *tight* selection to define the *relaxed tight* one are computed from the energy deposited in a few strips of the first compartment of the LAr EM calorimeter near the one with the largest deposit and are chosen since they have negligible correlations with the photon isolation. Due to the very small correlation (few %) between the track isolation and these discriminating variables, the background-photon track isolation efficiency is similar for photons satisfying *tight* or *relaxed tight* criteria. The differences between the track isolation efficiencies for background photons satisfying *tight* or *relaxed tight* criteria are included in the systematic uncertainties. The contamination from prompt photons in the background enriched samples is accounted for in this procedure by using as an additional input the fraction of signal events passing or failing the *relaxed tight* requirements, as determined from the prompt-photon simulation. The fraction of prompt photons in the background control samples decreases from about 20% to 1%, with increasing photon transverse momentum. The whole procedure is tested with a simulated sample of $\gamma +$jet and dijet events, and the difference between the true track isolation efficiency for background photons and the one estimated with this procedure is taken as a systematic uncertainty. An additional systematic uncertainty, due to the use of the prompt-photon simulation to estimate the fraction of signal photons in the background control regions, is estimated by re-calculating these fractions using alternative MC samples based on a detector simulation with a conservative estimate of additional material in front of the calorimeter. The typical total relative uncertainty in the background-photon track isolation efficiency is 2–4%.

**Fig. 6:**
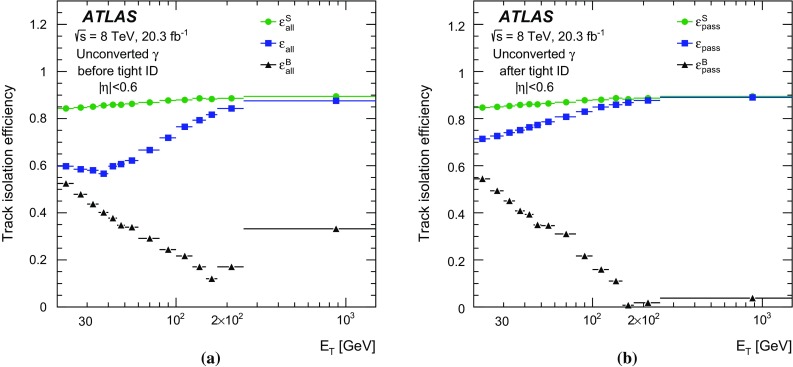
Track isolation efficiencies as a function of $E_{\text {T}}$ for unconverted prompt (*green circles*) and background (*black triangles*) photon candidates within $|\eta |< 0.6$ in **a** the inclusive sample or **b** passing *tight* identification requirements. The efficiencies are estimated combining the simulation and data control samples. The *blue square markers* show the track isolation efficiency for candidates selected in data

As an example, Fig. 6 shows the track isolation efficiencies as a function of $E_{\text {T}}$ for prompt and background unconverted photon candidates with $|\eta |<0.6$ in the ALL and PASS samples, as well as the fractions of all or *tight* photon candidates in data that satisfy the track isolation criteria. From these measurements the photon identification efficiency is derived, according to Eq. (3). The track isolation efficiency for prompt-photon candidates is essentially independent of the photon transverse momentum. For background candidates, the track isolation efficiency initially decreases with $E_{\text {T}}$, since candidates with larger $E_{\text {T}}$ are produced from more energetic jets, which are therefore characterised by a larger number of tracks near the photon candidate. At higher transverse energies, typically above 200 $\text {GeV}$, the boost of such tracks causes some of them to fall within the inner cone $(\Delta R<0.1)$ of the isolation cone around the photon and the isolation efficiency for background candidates therefore increases.

The total systematic uncertainty decreases with the transverse energy. It reaches 6% below 40 $\text {GeV}$, and amounts to 0.5–1% at higher $E_{\text {T}}$, where the contribution of this method is the most important.

The final result is obtained by multiplying the measured efficiency by a correction factor which takes into account the preselection of the sample using photon triggers, which already apply some loose requirements to the photon discriminating variables. The correction factor, equal to the ratio of the *tight* identification efficiency for all reconstructed photons to that for photons matching the trigger object that triggers the event, is obtained from a corrected simulation of photon+jet events. This correction is slightly lower than unity, by less than 1% for $E_{\text {T}} >50$ $\text {GeV}$ and by 2–3% for $E_{\text {T}}$ = 20 $\text {GeV}$. The systematic uncertainty from this correction is negligible compared to the other sources of uncertainty.

## Photon identification efficiency results at $\sqrt{s}=8$ $\text {TeV}$

### Efficiencies measured in data

**Fig. 7:**
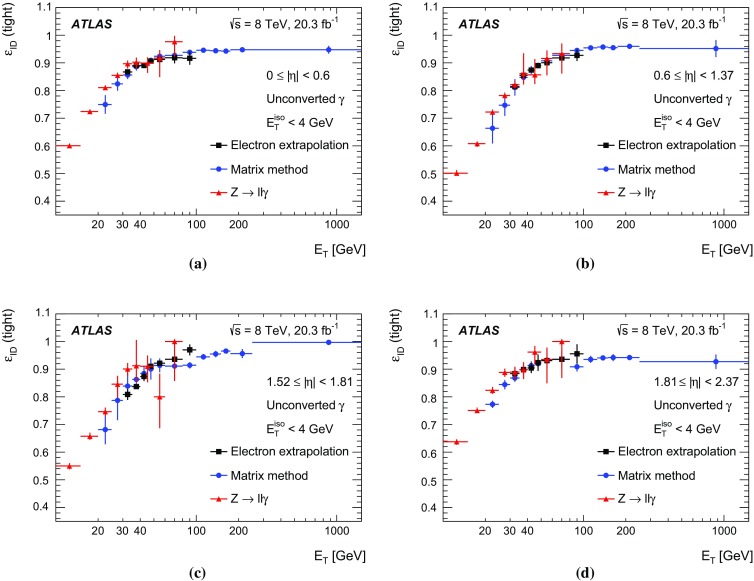
Comparison of the data-driven measurements of the identification efficiency for unconverted photons as a function of $E_{\text {T}}$ in the region $10~\text {GeV}< E_{\text {T}} < 1500~\text {GeV}$, for the four pseudorapidity intervals **a** $|\eta |<0.6$, **b** $0.6\le |\eta |<1.37$, **c** $1.52\le |\eta |<1.81$, and **d** $1.81\le |\eta |<2.37$. The *error bars* represent the sum in quadrature of the statistical and systematic uncertainties estimated in each method

**Fig. 8:**
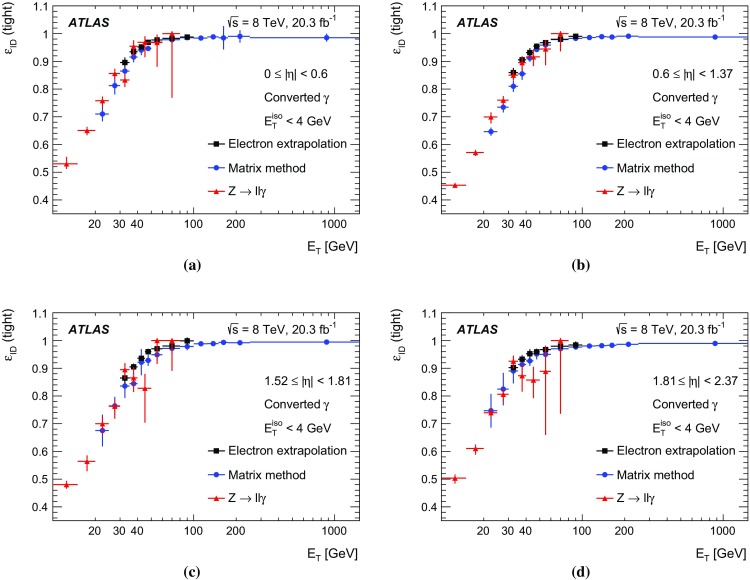
Comparison of the data-driven measurements of the identification efficiency for converted photons as a function of $E_{\text {T}}$ in the region $10~\text {GeV}< E_{\text {T}} < 1500~\text {GeV}$, for the four pseudorapidity intervals **a** $|\eta |<0.6$, **b** $0.6\le |\eta |<1.37$, **c** $1.52\le |\eta |<1.81$, and **d** $1.81\le |\eta |<2.37$. The *error bars* represent the quadratic sum of the statistical and systematic uncertainties estimated in each method

The identification efficiency measurements for $\sqrt{s}=8$ $\text {TeV}$ obtained from the three data-driven methods discussed in the previous section are compared in Figs. 7 and 8. The $Z\rightarrow ee\gamma$ and $Z\rightarrow \mu \mu \gamma$ results agree within uncertainties and are thus combined, following a procedure described in the next section, and only the combined values are shown in the figures. In a few $E_{\text {T}}$ bins in which the central values of the $Z\rightarrow ee\gamma$ and the $Z\rightarrow \mu \mu \gamma$ results differ by more than the combined uncertainty, the latter is increased to cover the full difference between the two results.

In the photon transverse momentum regions in which the different measurements overlap, the results from each method are consistent with each other within the uncertainties. Relatively large fluctuations of the radiative $Z$ decay measurements are seen, due to their large statistical uncertainties.

The photon identification efficiency increases from 50–65% (45–55%) for unconverted (converted) photons at $E_{\text {T}} \approx 10$ $\text {GeV}$ to 94–100% at $E_{\text {T}} \gtrsim 100$ $\text {GeV}$, and is larger than about 90% for $E_{\text {T}} >40$ $\text {GeV}$. The absolute uncertainty in the measured efficiency is around 1% (1.5%) for unconverted (converted) photons for $E_{\text {T}} <30$ $\text {GeV}$ and around 0.4–0.5% for both types of photons above 30 $\text {GeV}$ for the most precise method in a given bin.

### Comparison with the simulation

In this section the results of the data-driven efficiency measurements are compared to the identification efficiencies predicted in the simulation. The comparison is performed both before and after applying the shower shape corrections.

**Fig. 9:**
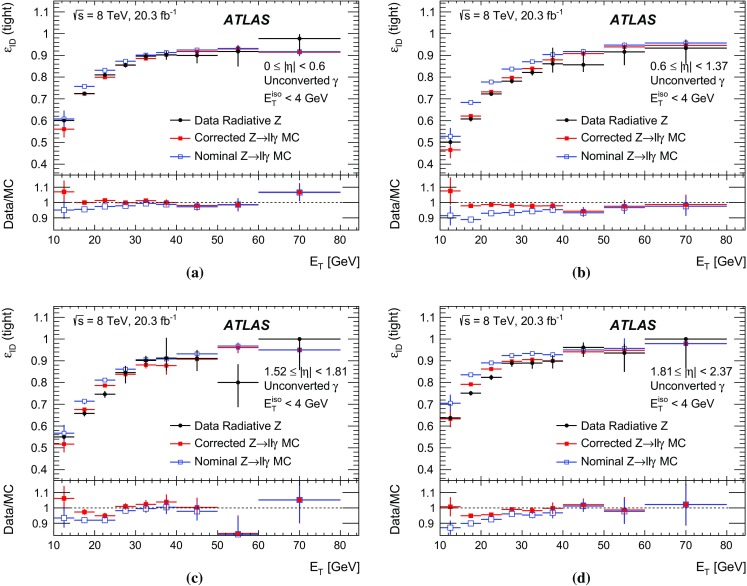
Comparison of the radiative $Z$ boson data-driven efficiency measurements of unconverted photons to the nominal and corrected $Z\rightarrow \ell \ell \gamma$ MC predictions as a function of $E_{\text {T}}$ in the region $10~\text {GeV}< E_{\text {T}} < 80~\text {GeV}$, for the four pseudorapidity intervals **a** $|\eta |<0.6$, **b** $0.6\le |\eta |<1.37$, **c** $1.52\le |\eta |<1.81$, and **d** $1.81\le |\eta |<2.37$. The *bottom panels* show the ratio of the data-driven results to the MC predictions (also called scale factors in the text). The *error bars* on the data points represent the quadratic sum of the statistical and systematic uncertainties. The *error bars* on the MC predictions correspond to the statistical uncertainty from the number of simulated events

**Fig. 10:**
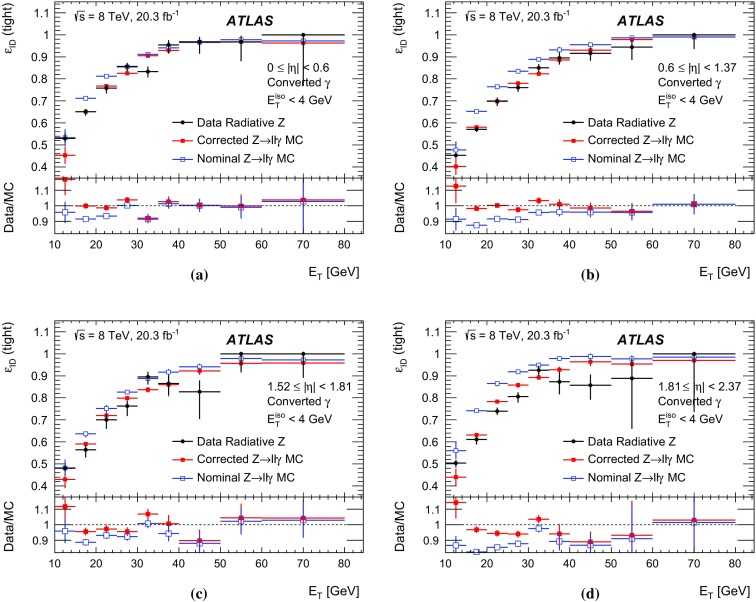
Comparison of the radiative $Z$ boson data-driven efficiency measurements of converted photons to the nominal and corrected $Z\rightarrow \ell \ell \gamma$ MC predictions as a function of $E_{\text {T}}$ in the region $10~\text {GeV}< E_{\text {T}} < 80~\text {GeV}$, for the four pseudorapidity intervals **a** $|\eta |<0.6$, **b** $0.6\le |\eta |<1.37$, **c** $1.52\le |\eta |<1.81$, and **d** $1.81\le |\eta |<2.37$. The *bottom panels* show the ratio of the data-driven results to the MC predictions (also called scale factors in the text). The *error bars* on the data points represent the quadratic sum of the statistical and systematic uncertainties. The *error bars* on the MC predictions correspond to the statistical uncertainty from the number of simulated events

**Fig. 11:**
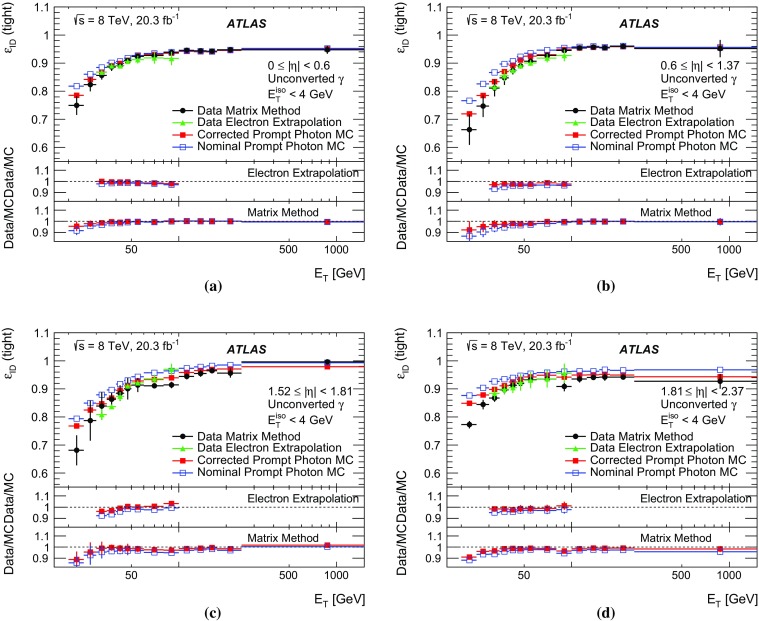
Comparison of the electron extrapolation and matrix method data-driven efficiency measurements of unconverted photons to the nominal and corrected prompt-photon+jet MC predictions as a function of $E_{\text {T}}$ in the region $20~\text {GeV}< E_{\text {T}} < 1500~\text {GeV}$, for the four pseudorapidity intervals **a** $|\eta |<0.6$, **b** $0.6\le |\eta |<1.37$, **c** $1.52\le |\eta |<1.81$, and **d** $1.81\le |\eta |<2.37$. The *bottom panels* show the ratio of the data-driven results to the MC predictions (also called scale factors in the text). The *error bars* on the data points represent the quadratic sum of the statistical and systematic uncertainties. The *error bars* on the MC predictions correspond to the statistical uncertainty from the number of simulated events

**Fig. 12:**
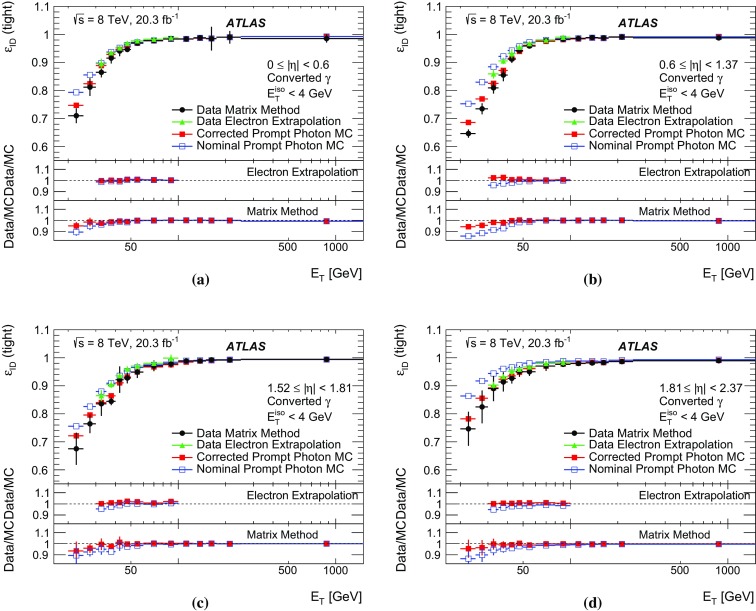
Comparison of the electron extrapolation and matrix method data-driven efficiency measurements of converted photons to the nominal and corrected prompt-photon+jet MC predictions as a function of $E_{\text {T}}$ in the region $20~\text {GeV}< E_{\text {T}} < 1500~\text {GeV}$, for the four pseudorapidity intervals **a** $|\eta |<0.6$, **b** $0.6\le |\eta |<1.37$, **c** $1.52\le |\eta |<1.81$, and **d** $1.81\le |\eta |<2.37$. The *bottom panels* show the ratio of the data-driven values to the MC predictions (also called scale factors in the text). The *error bars* on the data points represent the quadratic sum of the statistical and systematic uncertainties. The *error bars* on the MC predictions correspond to the statistical uncertainty from the number of simulated events

Prompt photons produced in photon+jet events have different kinematic distributions than photons originating in radiative *Z* boson decays. Moreover, some of the photons in $\gamma$+jet events – unlike those from *Z* boson decays – originate in parton fragmentation. Such photons have lower identification efficiency than the photons produced directly in the hard-scattering process, due to the energy deposited in the calorimeter by the hadrons produced almost collinearly with the photon in the fragmentation. After applying an isolation requirement, however, the fragmentation photons usually represent a small fraction of the selected sample, typically below 10% for low transverse momenta and rapidly decreasing to a few % with increasing $E_{\text {T}}$. The difference in identification efficiency between photons from radiative *Z* boson decays and from $\gamma$+jet events is thus expected to be small. To account for such a difference, the efficiency measured in data with the radiative $Z$ boson decay method is compared to the prediction from simulated $Z\rightarrow \ell \ell \gamma$ events (Figs. 9, 10), while the efficiency measured in data with the electron extrapolation and matrix methods is compared to the prediction from simulated photon+jet events (Figs. 11, 12).

The level of agreement among the different $\varepsilon _\mathrm {ID}$ values improves with increasing $E_{\text {T}}$: no significant difference is observed between the data-driven measurements and the nominal or corrected simulation for $E_{\text {T}} > 60$ $\text {GeV}$. At lower transverse momenta, the nominal simulation tends to overestimate the efficiency by up to 10–15%, as the electromagnetic showers from photons are typically narrower in the simulation than in data. In the same transverse momentum range, the corrected simulation agrees with the data-driven measurements within a few percent.

The remaining difference between the corrected simulation and the data-driven measurements is taken into account by computing data-to-MC efficiency ratios, also referred to as *scale factors* (SF). The data-to-MC efficiency ratios are computed separately for each method and then combined. The efficiencies from the $Z \rightarrow \ell \ell \gamma$ data control sample are divided by the prediction of the simulation of radiative photons from $Z$ boson decays, while the results from the other two methods are divided by the predictions of the photon+jet simulation. The data-to-MC efficiency ratios are shown in the bottom plots of Figs. 9, 10, 11 and 12 and are used to correct the predictions in the analyses using photons.

Because of their good agreement and the mostly independent data samples used, the data-to-MC efficiency ratios as a function of photon $E_{\text {T}}$ are combined into a single, more precise result in the overlapping regions. The combination is performed independently in the different pseudorapidity and transverse energy bins, using the Best Linear Unbiased Estimate (BLUE) method [45, 46]. The combined data-to-MC efficiency ratio SF is calculated as a linear combination of the input measurements, ${\mathrm {SF}}_i$, with coefficients $w_{i}$ that minimise the total uncertainty in the combined result. In the algorithm, both the statistical and systematic uncertainties, as well as the correlations of systematic sources between input measurements, are taken into account assuming that all uncertainties have Gaussian distributions. In practice, the quantity that is minimised is a $\chi ^2$ built from the various results and their statistical and systematic covariance matrices. Since the three measurements use different data samples and independent MC simulations, their systematic and statistical uncertainties are largely uncorrelated. The background-induced uncertainties in the $Z\rightarrow ee\gamma$ and $Z\rightarrow \mu \mu \gamma$ results, originating from the same background process (*Z*+jet events with a jet misreconstructed as a photon) and evaluated with the same method, are considered to be 100% correlated. The uncertainties in the results of the matrix method and the electron extrapolation method due to limited knowledge of the detector material in the simulation are also partially correlated, both being determined with alternative MC samples based on the same detector simulation with a conservative estimate of additional material in front of the calorimeter. The exact value of this correlation is difficult to estimate. However, it was checked by varying the amount of correlation that its effect on the final result is negligible.

After the combination, for each averaged scale factor SF, the $\chi ^2=\sum _{i=1}^{N} w_i({\mathrm {SF}}-{\mathrm {SF}}_i)^2$ is computed and compared to $N-1$, where *N* is the number of measurements included in the combined result for that point, and $N-1$ is the expectation value of $\chi ^2$ from a Gaussian distribution. Only a few bins among all photon $\eta$ and $E_{\text {T}}$ bins for unconverted and converted photons are found to have $\chi ^2/(N-1) >1$. These $\chi ^2$ values are smaller than 2.0, confirming that the different measurements are consistent. For the points with $\chi ^2/(N-1) > 1$, the error in the combined value, $\delta {\mathrm {SF}}$, is increased by a factor $S=\sqrt{\chi ^2/(N-1)}$, following the prescription in Ref. [47]. The combined data-to-MC efficiency ratios differ from one by as much as 10% at $E_{\text {T}}$ = 10 $\text {GeV}$ and by only a few percent above $E_{\text {T}}$ = 40 $\text {GeV}$.

A systematic uncertainty in the data-to-MC efficiency ratios is associated with the uncertainty in photon+jet simulation’s modelling of the fraction of photons emitted in the fragmentation of partons. In order to estimate the effect on the data-to-MC efficiency ratio, the number of fragmentation photons in the photon+jet MC sample is varied by ${\pm } 50\%$, and the maximum variation of the data-to-MC efficiency ratio is taken as an additional systematic uncertainty. This uncertainty decreases with increasing transverse momentum and is always below 0.5% and 0.7% for unconverted and converted photons, respectively. This uncertainty is also larger than the efficiency differences observed in the simulation between different event generators, which are thus not considered as a separate systematic uncertainty in the data-to-MC efficiency ratios.

The effect of the isolation requirement on the data-to-MC efficiency ratios is checked by varying it between 3 and 7 $\text {GeV}$ and recomputing the data-to-MC efficiency ratios using $Z$ boson radiative decays. The study is performed in different regions of pseudorapidity and integrated over $E_{\text {T}}$ to reduce statistical fluctuations. The deviation of the alternative data-to-MC efficiency ratios from the nominal value is typically 0.5% and always lower than 1.2%, almost independent of pseudorapidity. This deviation is thus considered as an additional uncertainty and added in quadrature in ATLAS measurements with final-state photons to which an isolation requirement different from ${E_{\mathrm {T}}^{\mathrm {iso}}}<4$ $\text {GeV}$ is applied.

**Fig. 13:**
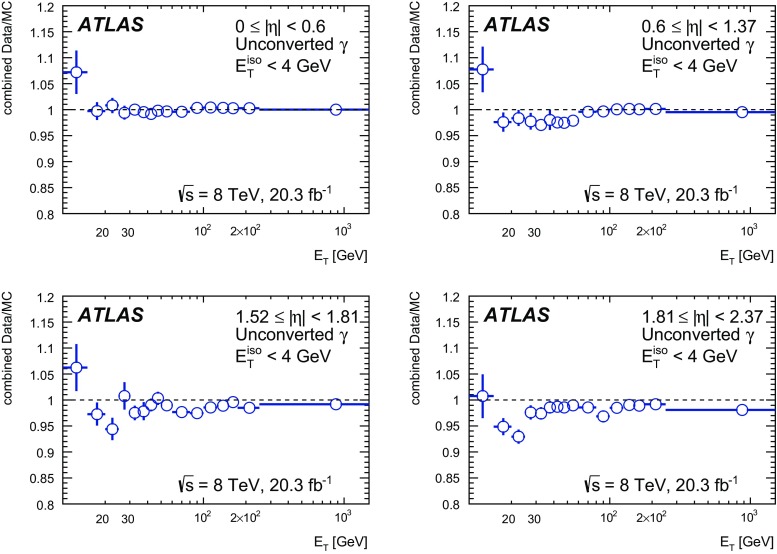
Combined data-to-MC efficiency ratios (SF) of unconverted photons in the region $10~\text {GeV}< E_{\text {T}} < 1500~\text {GeV}$

**Fig. 14:**
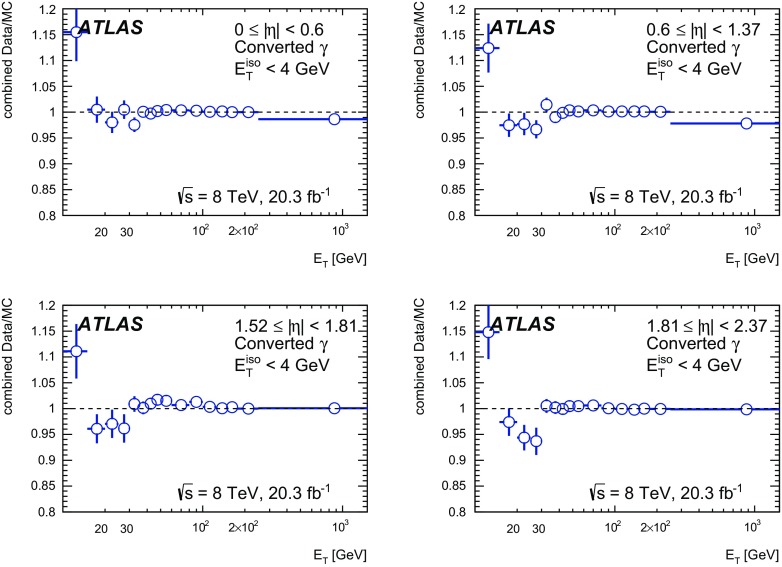
Combined data-to-MC efficiency ratios (SF) of converted photons in the region $10~\text {GeV}< E_{\text {T}} < 1500~\text {GeV}$

The combined data-to-MC efficiency ratios with their total uncertainties are shown as a function of $E_{\text {T}}$ in Figs. 13 and 14. In the low transverse energy region these ratios decrease from values higher than one to values smaller than one because the data and MC efficiency curves cross between 10 and 20 $\text {GeV}$, as can be seen in Figs. 9 and 10. The change of shape at $E_{\text {T}}$ = 30 $\text {GeV}$ can be explained by the fact that the electron extrapolation method starts entering the combination, changing the central values but also decreasing the uncertainties.

The total uncertainty in the data-to-MC efficiency ratio is 1.4–4.5% (1.7–5.6%) for unconverted (converted) photons for $10~\text {GeV} <E_{\text {T}} < 30~\text {GeV}$, it decreases to 0.2–2.0% (0.2–1.5%) for $30~\text {GeV} <E_{\text {T}} < 100~\text {GeV}$, and it further decreases to 0.2–0.8% (0.2–0.5%) for higher transverse momenta. The ${\approx } 5\%$ uncertainty at low transverse momenta is due to the systematic uncertainty affecting the measurement with radiative *Z* boson decays for $10~\text {GeV} <E_{\text {T}} <15$ $\text {GeV}$. Above 15 $\text {GeV}$ the total uncertainty is below 2.5% (3.0%) for unconverted (converted) photons. A summary of the contributions to the final uncertainty on the data-to-MC efficiency ratios of the different sources of uncertainties described in Sect. 5 is given in Table 2. The background systematic uncertainties correspond to the background subtraction done in the three methods. The material uncertainty comes from limited knowledge of the material upstream of the calorimeter which affects the shower-shape description for the electron extrapolation method (Sect. 5.2) and the track isolation efficiency for the matrix method (Sect. 5.3). The non-closure test uncertainty of the Smirnov transform appears only in the electron extrapolation method (Sect. 5.2).

**Table 2:** Ranges of total uncertainty on the data-to-MC photon identification efficiency ratios and breakdown of the different sources of uncertainty for unconverted and converted photons, in three bins of transverse energy, giving the minimum and maximum values in the four pseudorapidity regions

	10–30 $\text {GeV}$	30–100 $\text {GeV}$	100–1500 $\text {GeV}$
Unconverted $\gamma$			
Total uncertainty	1.4–4.5%	0.2–2.0%	0.2–0.8%
Statistical uncertainty	0.5–2.0%	0.1–0.7%	0.1–0.4%
Total systematic uncertainty	1.0–4.1%	0.1–1.2%	0.1–0.8%
Background uncertainty	0.6–1.3%	0.0–0.8%	0.0–0.7%
Material uncertainty	0.0–0.8%	0.0–1.1%	0.0–0.8%
Non closure	0.0%	0.0–0.9%	0.0%
Converted $\gamma$			
Total uncertainty	1.7–5.6%	0.2–1.5%	0.2–0.5%
Statistical uncertainty	0.9–3.2%	0.1–0.6%	0.1–0.4%
Total systematic uncertainty	1.4–4.3%	0.2–1.4%	0.1–0.5%
Background uncertainty	0.7–1.7%	0.0–0.6%	0.0–0.4%
Material uncertainty	0.0–1.3%	0.0–1.0%	0.0–0.5%
Non closure	0.0%	0.0–0.9%	0.0%

In multi-photon processes, such as Higgs boson decays to two photons, a per-event efficiency correction to the simulated events is computed by applying scale factors to each of the photons in the event. The associated uncertainty depends on the correlation between SF uncertainties in different regions of photon $|\eta |$ and $E_{\text {T}}$, and for converted and unconverted photons. Among the systematic uncertainties considered in the analysis, the impact of correlations is found to be negligible in all cases but one, that of the uncertainty in the background level in the matrix method determination (see Sect. 5.3). Its contribution to the SF uncertainty is conservatively assumed to be fully correlated across all regions of $|\eta |$ and $E_{\text {T}}$ and between converted and unconverted photons, while the rest of the SF uncertainty is assumed to be uncorrelated. The correlated and uncorrelated components of the uncertainty in each region are then propagated to the per-event uncertainty using a toy-experiment technique.

## Photon identification efficiency at $\sqrt{s}=7$ $\text {TeV}$

As described in Sect. 3.2, photon identification in the analysis of 7 $\text {TeV}$ data relies on the same cut-based algorithms used for the 8 $\text {TeV}$ data, with different thresholds. Such thresholds were first determined using simulated samples prior to the 2010 data-taking and then loosened in order to reduce the observed inefficiency and the systematic uncertainties arising from the differences found between the distributions of the discriminating variables in data and in the simulation.

The efficiency of the identification algorithms used for the analysis of the 7 $\text {TeV}$ data is measured with the same techniques described in Sect. 5. Small differences between the 7 and 8 $\text {TeV}$ measurements concern the simulated samples that were used, and the criteria used to select the data control samples. The 7 $\text {TeV}$ simulations are based on a different detector material model, as described in Sect. 4; the number of simulated pile-up interactions and the correction factors for the lepton efficiency and momentum scale and resolution also differ from those of the 8 $\text {TeV}$ study, as do the lepton triggers and the algorithms used to identify the leptons in data. Due to the smaller number of events, the 7 $\text {TeV}$ measurements cover a narrower transverse momentum range, $20~\text {GeV}<E_{\text {T}} <250$ $\text {GeV}$. The nominal efficiency is measured with respect to photons having a calorimeter isolation transverse energy lower than 5 $\text {GeV}$, a typical requirement used in 7 $\text {TeV}$ ATLAS measurements. The isolation energy is computed using all the calorimeter cells in a cone of $\Delta R=0.4$ around the photon and corrected for pile-up and the photon energy.

**Fig. 15:**
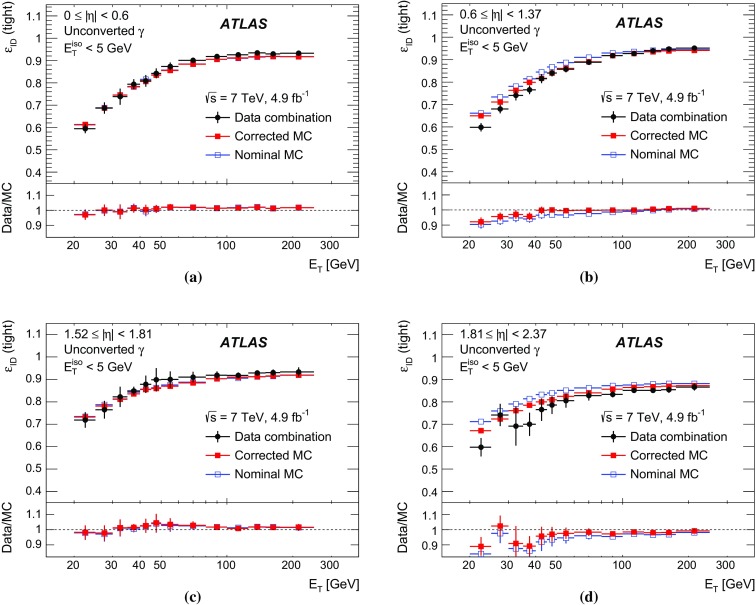
Comparison between the identification efficiency ${\varepsilon _\mathrm {ID}}$ of unconverted photon candidates in $\sqrt{s} = 7~\text {TeV}$ data and in the nominal and corrected MC predictions in the region $20~\text {GeV}< E_{\text {T}} < 250~\text {GeV}$, for the four pseudorapidity intervals **a** $|\eta |<0.6$, **b** $0.6\le |\eta |<1.37$, **c** $1.52\le |\eta |<1.81$, and **d** $1.81\le |\eta |<2.37$. The *black error bars* correspond to the sum in quadrature of the statistical and systematic uncertainties estimated for the combination of the data-driven methods. Only the statistical uncertainties are shown for the MC predictions. The *bottom panels* show the ratio of the data-driven results to the nominal and corrected MC predictions

**Fig. 16:**
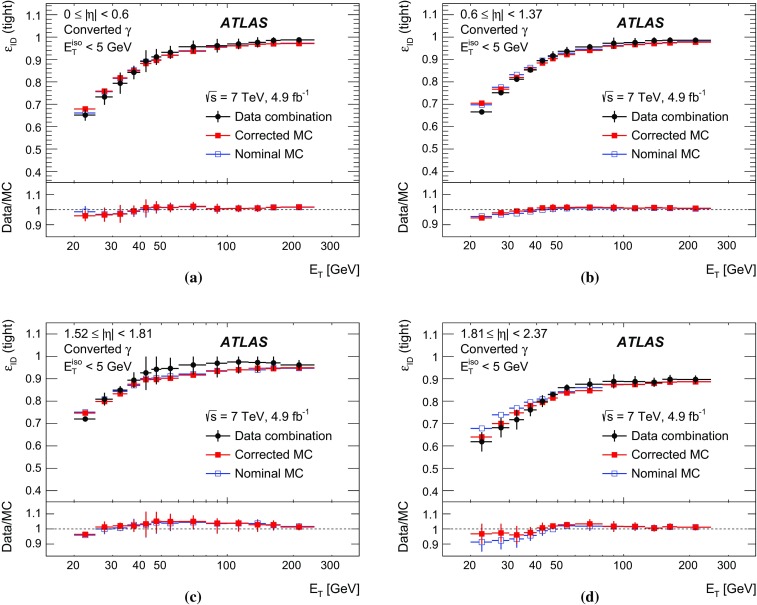
Comparison between the identification efficiency ${\varepsilon _\mathrm {ID}}$ of converted photon candidates in $\sqrt{s}=7~\text {TeV}$ data and in the nominal and corrected MC predictions in the region $20~\text {GeV}< E_{\text {T}} < 250~\text {GeV}$, for the four pseudorapidity intervals **a** $|\eta |<0.6$, **b** $0.6\le |\eta |<1.37$, **c** $1.52\le |\eta |<1.81$, and **d** $1.81\le |\eta |<2.37$. The *black errors bars* correspond to the sum in quadrature of the statistical and systematic uncertainties estimated for the combination of the data-driven methods. Only the statistical uncertainties are shown for the MC predictions. The *bottom panels* show the ratio of the data-driven results to MC predictions (also called scale factors in the text)

The number of selected candidates is 12000 in the $Z \rightarrow \ell \ell \gamma$ study, $1.8\times 10^6$ in the $Z \rightarrow ee$ one, and $1.5\times 10^7$ in the measurement with the matrix method. All data-driven measurements are combined using the same procedure described in Sect. 6.2 for the scale factors, and then compared to a simulation of prompt-photon+jet events. In the combination, the differences between the efficiencies of photons from radiative *Z* boson decays and of photons from $\gamma +$jet events mentioned in Sect. 6.2 are neglected. Such differences after the photon isolation requirement are estimated to be much smaller than the uncertainties of the measurements performed with the $\sqrt{s}=7$ TeV data. The combined efficiency measurements for the cut-based identification algorithms at $\sqrt{s}=7$ $\text {TeV}$ are shown in Figs. 15 and 16. The identification efficiency increases from 60–70% for $E_{\text {T}}$ = 20 $\text {GeV}$ to 87–95% (90–99%) for $E_{\text {T}}\ > 100~\text {GeV}$ for unconverted (converted) photons. The uncertainty in the efficiency and on the data-to-MC efficiency ratios decreases from 3–10% at low $E_{\text {T}}$ to about 0.5–5% for $E_{\text {T}}\ > 100~\text {GeV}$, being typically larger at higher $|\eta |$.

In the search of the Higgs boson decays to diphoton final states with 7 $\text {TeV}$ data [23], an alternative photon identification algorithm based on an artificial neural network (NN) was used. The neural network uses as input the same discriminating variables exploited by the cut-based selection. Multi-layer perceptrons are implemented with the Toolkit for Multivariate Data Analysis [48], using 13 nodes in a single hidden layer. Separate networks are optimised along bins of photon pseudorapidity and transverse momentum. Different networks are created for photons that are reconstructed as unconverted, single-track converted and double-track converted, due to their different distributions of the discriminating variables. The final identification is performed by requiring the output discriminant to be larger than a certain threshold, tuned to reproduce the background photon rejection of the cut-based algorithm. For the training of the NN, simulated signal events and jet-enriched data are used. In the simulation, the discriminating variables are corrected for the average differences observed with respect to the data. For the NN-based photon identification algorithm, the efficiency increases from 85–90% for $E_{\text {T}}$ $=$ 20 $\text {GeV}$ to about 97% (99%) for $E_{\text {T}} >100~\text {GeV}$ for unconverted (converted) photon candidates, with uncertainties varying between 4 and 7%.

## Dependence of the photon identification efficiency on pile-up

The dependence of the identification efficiency and of the data/MC efficiency scale factors on pile-up was investigated with both 7 and 8 $\text {TeV}$ data. The efficiencies are measured as a function of the number of reconstructed primary vertex candidates with at least three associated tracks, $N_{\mathrm {PV}}$, a quantity which is highly correlated to $\mu$, the expected number of interactions per bunch crossing.

In 2012 *pp* collisions, $\mu$ was typically between 1 and 40, with an average value of 21. In the range $10~\text {GeV}<E_{\text {T}} <30$ $\text {GeV}$ the pile-up dependence of the $\sqrt{s}=8$ $\text {TeV}$ identification efficiency is measured using $Z$ boson radiative decays, integrating over the photon pseudorapidity distribution because of the small size of the sample. For higher transverse momenta the dependence is measured using the results obtained with the electron extrapolation method, in four $|\eta |$ bins.

In $\sqrt{s}=7$ $\text {TeV}$ *pp* collisions, the pile-up dependence is measured using the results obtained the matrix method, in four $|\eta |$ bins, integrated over the $E_{\text {T}} >20$ $\text {GeV}$ range.

**Fig. 17:**
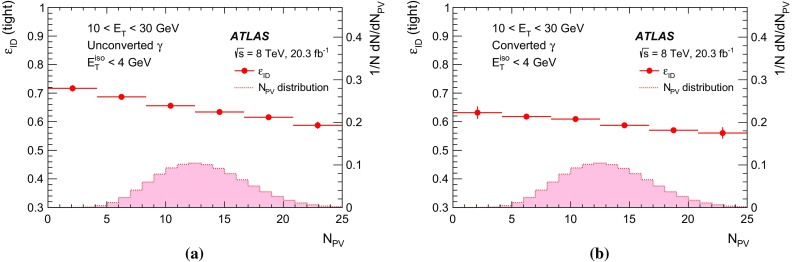
Efficiency (*red dots*) of **a** unconverted and **b** converted photons candidates as a function of the number $N_{\mathrm {PV}}$ of reconstructed primary vertices, measured in 2012 data from radiative *Z* boson decays. The measurements are integrated in pseudorapidity and in the transverse momentum range $10~\text {GeV}< E_{\text {T}}\ <30~\text {GeV}$. The red histograms indicate the $N_{\mathrm {PV}}$ distribution in 2012 data

**Fig. 18:**
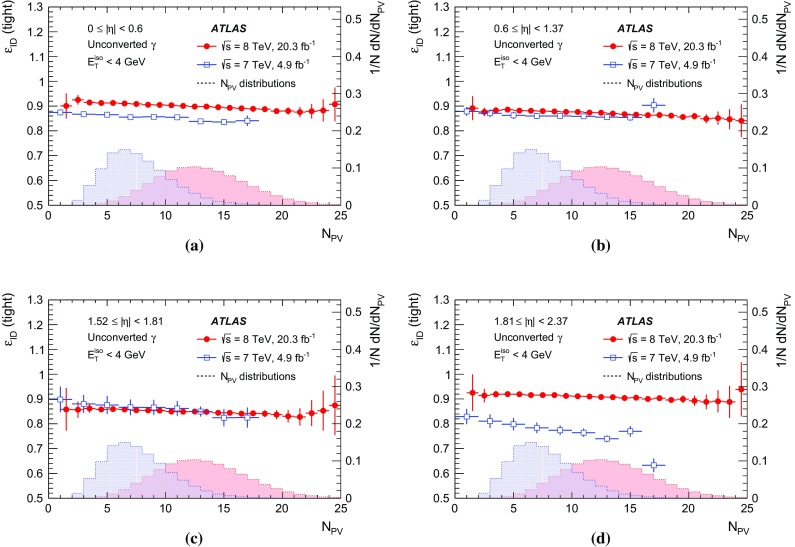
Comparison of data-driven efficiency measurements for unconverted photons performed with the 2011 (*blue squares*) and 2012 (*red circles*) datasets as a function of the number $N_{\mathrm {PV}}$ of reconstructed primary vertex candidates, for the four pseudorapidity intervals **a** $|\eta |<0.6$, **b** $0.6\le |\eta |<1.37$, **c** $1.52\le |\eta |<1.81$, and **d** $1.81\le |\eta |<2.37$. The 2011 measurements are performed with the matrix method for photons with $E_{\text {T}}\ > 20~\text {GeV}$ and the 2012 measurements with the electron extrapolation method for photons with $E_{\text {T}}\ > 30~\text {GeV}$. The two (*blue*/*red*) histograms indicate the $N_{\mathrm {PV}}$ distribution in 2011/2012 data

**Fig. 19:**
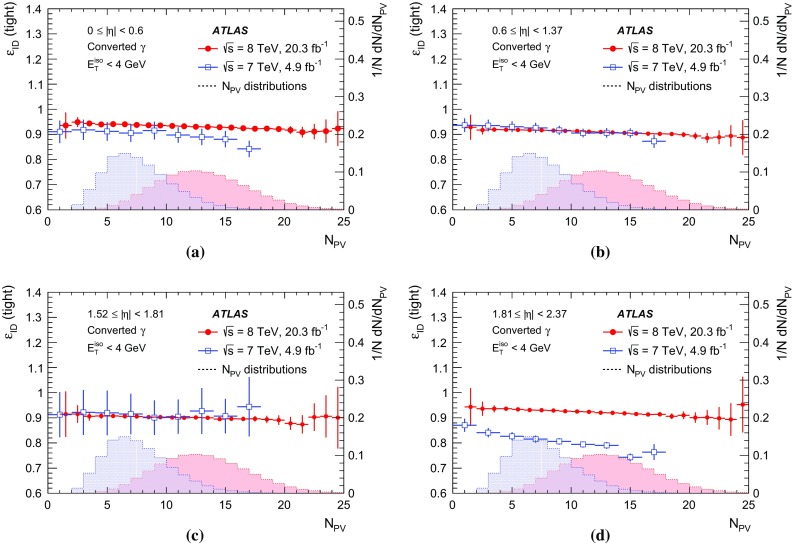
Comparison of data-driven efficiency measurements for converted photons performed with the 2011 (*blue squares*) and 2012 (*red circles*) datasets as a function of the number $N_{\mathrm {PV}}$ of reconstructed primary vertex candidates, for the four pseudorapidity intervals **a** $|\eta |<0.6$, **b** $0.6\le |\eta |<1.37$, **c** $1.52\le |\eta |<1.81$, and **d** $1.81\le |\eta |<2.37$. The 2011 measurements are performed with the matrix method for photons with $E_{\text {T}}\ > 20~\text {GeV}$ and the 2012 measurements with the electron extrapolation method for photons with $E_{\text {T}}\ > 30~\text {GeV}$. The two (*blue*/*red*) histograms indicate the $N_{\mathrm {PV}}$ distribution in 2011/2012 data

The results of the data measurements are shown in Figs. 17, 18 and 19. The efficiency variation with $N_{\mathrm {PV}}$ in $\sqrt{s}=8$ $\text {TeV}$ data for $E_{\text {T}} <30$ $\text {GeV}$ is shown in Fig. 17. The variation is rather large, up to 15% in the range $0<N_{\mathrm {PV}}\le 20$ (corresponding to about $0<\mu \le 30$). The efficiency variation with $N_{\mathrm {PV}}$ in $\sqrt{s}=8$ (7) $\text {TeV}$ data for $E_{\text {T}} >30$ (20) $\text {GeV}$ is shown in Figs. 18 and 19. In the 8 $\text {TeV}$ data the efficiency dependence on pile-up for $E_{\text {T}} >30$ $\text {GeV}$ is similar in the pseudorapidity intervals that have been studied, with a decrease of about 3–4% when $N_{\mathrm {PV}}$ increases from 1 to 20. The pile-up dependence of the photon identification efficiency is smaller in 8 $\text {TeV}$ data than in 7 $\text {TeV}$ data, since the photon identification criteria were specifically re-optimised to be less sensitive to pile-up before the start of the 8 $\text {TeV}$ data taking.

To further study the pile-up dependence of the efficiency at high photon transverse momenta, the $\sqrt{s}=8$ $\text {TeV}$ measurements with the electron extrapolation have been repeated using only electron probes with $E_{\text {T}} >45$ $\text {GeV}$. The efficiency for $E_{\text {T}} > 45~\text {GeV}$ photons decreases by only 1–3% when $N_{\mathrm {PV}}$ increases from 1 to 20.

The pile-up dependence of the efficiency in data is compared to the prediction of the simulation by calculating the data-to-MC efficiency ratios as a function of the number of reconstructed primary vertex candidates $N_{\mathrm {PV}}$. The pile-up dependence of the data-to-MC efficiency ratios is assessed through a linear fit of the efficiency ratios as a function of $N_{\mathrm {PV}}$. The slopes of these fits are always consistent with zero within the uncertainties, which are of the order of 0.2%. Therefore, while the efficiency itself varies significantly as a function of $N_{\mathrm {PV}}$, the dependence of the data-to-MC efficiency ratios on $N_{\mathrm {PV}}$ in the range $0<N_{\mathrm {PV}}\le 26$ (corresponding to about $0<\mu \le 40$) is compatible with zero. This observation suggests that the simulation correctly models the effect of pile-up on the distributions of the discriminating variables.

## Conclusion

The efficiency ${\varepsilon _\mathrm {ID}}$ of the algorithms used by ATLAS to identify photons during the LHC Run 1 has been measured from *pp* collision data using three independent methods in different photon $E_{\text {T}}$ ranges. The three measurements agree within their uncertainties in the overlapping $E_{\text {T}}$ ranges, and are combined.

For the data taken in 2011, 4.9 fb$^{-1}$ at $\sqrt{s}=7$ $\text {TeV}$, the efficiency of the cut-based identification algorithm increases from 60–70% at $E_{\text {T}} =20$ $\text {GeV}$ up to 87–95% (90–99%) at $E_{\text {T}} >100$ $\text {GeV}$ for unconverted (converted) photons. With an optimised neural network this efficiency increases from 85–90% at $E_{\text {T}} =20$ $\text {GeV}$ to about 97% (99%) at $E_{\text {T}} >100$ $\text {GeV}$ for unconverted (converted) photon candidates for a similar background rejection. For the data taken in 2012, 20.3 fb$^{-1}$ at $\sqrt{s}=8$ $\text {TeV}$, the efficiency of a re-optimised cut-based photon identification algorithm increases from 50–65% (45–55%) for unconverted (converted) photons at $E_{\text {T}} =10$ $\text {GeV}$ to 95–100% at $E_{\text {T}} > 100$ $\text {GeV}$, being larger than ${\approx } 90\%$ for $E_{\text {T}} >40$ $\text {GeV}$.

The nominal MC simulation of prompt photons in ATLAS predicts significantly higher identification efficiency values than those measured in some regions of the phase space, particularly at low $E_{\text {T}}$. A simulation with shower shapes corrected for the average shifts observed with respect to the data describes the values of ${\varepsilon _\mathrm {ID}}$ better in the entire $E_{\text {T}}$ and $\eta$ range accessible by the data-driven methods. The residual difference between the efficiencies in data and in the corrected simulation are taken into account by computing data-to-MC efficiency scale factors. These factors differ from one by up to 10% at $E_{\text {T}} =10$ $\text {GeV}$ and by only a few percents above $E_{\text {T}} = 40$ $\text {GeV}$, with an uncertainty decreasing from 1.4–4.5% (1.7–5.6%) at $E_{\text {T}} =10$ $\text {GeV}$ for unconverted (converted) photons to 0.2–0.8% (0.2–0.5%) at high $E_{\text {T}}$ for $\sqrt{s}=8$ $\text {TeV}$. The uncertainties are slightly larger for $\sqrt{s}=7$ $\text {TeV}$ data due to the smaller size of the control samples.

## Definition of the photon identification discriminating variables

In this Appendix, the quantities used in the selection of photon candidates, based on the reconstructed energy deposits in the ATLAS calorimeters, are summarised.

- **Leakage in the hadronic calorimeter** The following discriminating variables are defined, based on the energy deposited in the hadronic calorimeter:
  - *Normalised hadronic leakage*
    4 $$\begin{aligned} R_\mathrm {had}= \frac{E_{\text {T}} ^\mathrm {had}}{E_{\text {T}}} \end{aligned}$$
    is the transverse energy $E_{\text {T}} ^\mathrm {had}$ deposited in cells of the hadronic calorimeter whose centre is in a window $\Delta \eta \times \Delta \phi$ = 0.24 $\times$ 0.24 behind the photon cluster, normalised to the total transverse energy $E_{\text {T}}$ of the photon candidate.
  - *Normalised hadronic leakage in first layer*
    5 $$\begin{aligned} R_\mathrm {had_{1}}= \frac{E_{\text {T}} ^\mathrm {had,1}}{E_{\text {T}}} \end{aligned}$$
    is the transverse energy $E_{\text {T}} ^\mathrm {had,1}$ deposited in cells of the first layer of the hadronic calorimeter whose centre is in a window $\Delta \eta \times \Delta \phi$ = 0.24 $\times$ 0.24 behind the photon cluster, normalised to the total transverse energy $E_{\text {T}}$ of the photon candidate.
  The $R_\mathrm {had}$ variable is used in the selection of photon candidates with pseudorapidity $|\eta |$ between 0.8 and 1.37 while the $R_\mathrm {had_{1}}$ variable is used otherwise.
- **Variables using the second (“middle”) layer of the electromagnetic calorimeter** The discriminating variables based on the energy deposited in the second layer of the electromagnetic calorimeter are the following:
  - *Middle* $\eta$ *energy ratio*
    6 $$\begin{aligned} R_{\eta }= \frac{E_{3\times 7}^{S2}}{E_{7\times 7}^{S2}} \end{aligned}$$
    is the ratio of the sum $E_{3\times 7}^{S2}$ of the energies of the second-layer cells of the electromagnetic calorimeter contained in a 3$\times$7 rectangle in $\eta \times \phi$ measured in cell units ($0.025\times 0.0245$), to the sum $E_{7\times 7}^{S2}$ of the energies in a 7$\times$7 rectangle, both centred around the cluster seed.
  - *Middle* $\phi$ *energy ratio*
    7 $$\begin{aligned} R_{\phi }= \frac{E_{3\times 3}^{S2}}{E_{3\times 7}^{S2}} \end{aligned}$$
    is defined similarly to $R_{\eta }$. $R_{\phi }$ behaves very differently for unconverted and converted photons, since the electrons and positrons generated by the latter bend in different directions in $\phi$ because of the solenoid’s magnetic field, producing larger showers in the $\phi$ direction than the unconverted photons.
  - *Middle lateral width*
    8 $$\begin{aligned} w_{\eta _2}= \sqrt{ \frac{\sum E_i \eta _i^2}{\sum E_i} - \left( \frac{\sum E_i \eta _i}{\sum E_i} \right) ^2} \end{aligned}$$
    where $E_{i}$ is the energy deposit in each cell, and $\eta _i$ is the actual $\eta$ position of the cell, measures the shower’s lateral width in the second layer of the electromagnetic calorimeter, using all cells in a window $\eta \times \phi = 3 \times 5$ measured in cell units.
- **Variables using the first (“front”) layer of the electromagnetic calorimeter** The discriminating variables based on the energy deposited in the first layer of the electromagnetic calorimeter are the following:
  - *Front side energy ratio*
    9 $$\begin{aligned} F_{\mathrm {side}}= \frac{E(\pm 3) - E(\pm 1)}{E(\pm 1)} \end{aligned}$$
    measures the lateral containment of the shower, along the $\eta$ direction. $E(\pm n)$ is the energy in the $\pm n$ strip cells around the one with the largest energy.
  - *Front lateral width* (3 *strips*)
    10 $$\begin{aligned} w_{s\,3}= \sqrt{\frac{\sum E_i (i-i_{\mathrm {max}})^2}{\sum E_i}} \end{aligned}$$
    measures the shower width along $\eta$ in the first layer of the electromagnetic calorimeter, using a total of three strip cells centred on the largest energy deposit. The index *i* is the strip identification number, $i_{\mathrm {max}}$ identifies the strip cells with the greatest energy, and $E_i$ is the energy deposit in each strip cell.
  - *Front lateral width* (*total*) $w_{s\,\mathrm {tot}}$ measures the shower width along $\eta$ in the first layer of the electromagnetic calorimeter using all cells in a window $\Delta \eta \times \Delta \phi = 0.0625 \times 0.196$, corresponding approximately to $20\times 2$ strip cells in $\eta \times \phi$, and is computed as $w_{s\,3}$.
  - *Front second maximum energy difference*
    11 $$\begin{aligned} \Delta {}E= \left[ E_{2^{\mathrm {nd}}{\mathrm {max}}}^{S1} - E_{\mathrm {min}}^{S1} \right] \end{aligned}$$
    is the difference between the energy of the strip cell with the second largest energy $E_{2^{\mathrm {nd}} {\mathrm {max}}}^{S1}$, and the energy in the strip cell with the lowest energy found between the largest and the second largest energy $E_{\mathrm {min}}^{S1}$ ($\Delta {}E=0$ when there is no second maximum).
  - *Front maxima relative energy ratio*
    12 $$\begin{aligned} E_{\mathrm {ratio}}=\frac{E_{1^{\mathrm {st}}\,{\mathrm {max}}}^{S1}-E_{2^{\mathrm {nd}}\, {\mathrm {max}}}^{S1}}{E_{1^{\mathrm {st}}\,{\mathrm {max}}}^{S1}+E_{2^{\mathrm {nd}} \,{\mathrm {max}}}^{S1}} \end{aligned}$$
    measures the relative difference between the energy of the strip cell with the largest energy $E_{1^{\mathrm {st}}\,{\mathrm {max}}}^{S1}$ and the energy in the strip cell with second largest energy $E_{2^{\mathrm {nd}}\,{\mathrm {max}}}^{S1}$ ($E_{\mathrm {ratio}}=1$ when there is no second maximum).

## Footnotes

1 ATLAS uses a right-handed coordinate system with its origin at the nominal interaction point (IP) in the centre of the detector and the *z*-axis along the beam pipe. The *x*-axis points from the IP to the centre of the LHC ring, and the *y*-axis points upward. Cylindrical coordinates $(r,\phi )$ are used in the transverse plane, $\phi$ being the azimuthal angle around the beam pipe. The pseudorapidity is defined in terms of the polar angle $\theta$ as $\eta =-\ln \tan (\theta /2)$. The photon transverse momentum is $E_{\text {T}} =E/\cosh (\eta )$, where *E* is its energy.

2 About 6.3% of the *b*-layer modules were disabled at the end of Run 1 due to individual module failures like low-voltage or high-voltage powering faults or data transmission faults. During the shutdown following the end of Run 1, repairs reduced the *b*-layer fault fraction to 1.4%

3 For converted photon candidates, the energy calibration procedure uses the following as additional inputs: (i) $p_{\text {T}}/E_{\text {T}}$ and the momentum balance of the two conversion tracks if both tracks are reconstructed by the silicon detectors, and (ii) the conversion radius for photon candidates with transverse momentum above 3 $\text {GeV}$.
